# Graphene transistors for interfacing with cells: towards a deeper understanding of liquid gating and sensitivity

**DOI:** 10.1038/s41598-017-06906-5

**Published:** 2017-07-27

**Authors:** Dmitry Kireev, Max Brambach, Silke Seyock, Vanessa Maybeck, Wangyang Fu, Bernhard Wolfrum, Andreas Offenhäusser

**Affiliations:** 1 Institute of Bioelectronics (PGI-8/ICS-8), Forschungszentrum Jülich, 52425 Jülich, Germany; 2 Faculty of Science, Leiden Institute of Chemistry, Supramolecular & Biomaterials Chemistry, Gorlaeus Laboratories, Einsteinweg 55, 2333 CC Leiden, The Netherlands; 3 Neuroelectronics, Munich School of Bioengineering, Department of Electrical and Computer Engineering, Technical University of Munich (TUM) & BCCN Munich, Boltzmannstr. 11, 85748 Garching, Germany

## Abstract

This work is focused on the fabrication and analysis of graphene-based, solution-gated field effect transistor arrays (GFETs) on a large scale for bioelectronic measurements. The GFETs fabricated on different substrates, with a variety of gate geometries (width/length) of the graphene channel, reveal a linear relation between the transconductance and the width/length ratio. The area normalised electrolyte-gated transconductance is in the range of 1–2 mS·V^−1^·□ and does not strongly depend on the substrate. Influence of the ionic strength on the transistor performance is also investigated. Double contacts are found to decrease the effective resistance and the transfer length, but do not improve the transconductance. An electrochemical annealing/cleaning effect is investigated and proposed to originate from the out-of-plane gate leakage current. The devices are used as a proof-of-concept for bioelectronic sensors, recording external potentials from both: *ex vivo* heart tissue and *in vitro* cardiomyocyte-like HL-1 cells. The recordings show distinguishable action potentials with a signal to noise ratio over 14 from *ex vivo* tissue and over 6 from the cardiac-like cell line *in vitro*. Furthermore, *in vitro* neuronal signals are recorded by the graphene transistors with distinguishable bursting for the first time.

## Introduction

In the field of bioelectronics, graphene is a promising candidate for very efficient, flexible, biocompatible and implantable sensors^1–3^. Graphene field effect transistors (GFETs) are the main focus of the work. In general, transistors are very interesting for bioelectronics, since when compared to microelectrode arrays (MEAs)^4^ they are active elements and are therefore more functional and tunable. Graphene transistors have already been shown to be extremely sensitive to changes in the gate potential in a liquid environment^5^. Moreover, it is possible to decrease the device’s size without impairing its performance (if the W/L ratio is preserved), which is a great advantage when compared to classical microelectrode arrays (MEAs)^4^. Additionally, even large areas of graphene have been proven to be both biocompatible and cytocompatible^6, 7^.

Additionally, in order to conduct good quality extracellular measurements reproduciblythe devices need to be identical or close to identical. However, up to now most fabrication routes for graphene-based bioelectronics are at an early development stage where devices are processed individually or in small arrays comprising only of a few devices and fabricated on a chip-scale^5, 8, 9^.

In recent years there have been many attempts to scale up the single-device processing to wafer-scale fabrication; some are still focused on epitaxially grown graphene^10, 11^, while some have attempted using chemical vapor deposition (CVD) graphene for the wafer scale fabrication of devices^12–14^. One of the main problems in this regard is the quality of CVD-grown graphene^15^. However, up to now, CVD graphene can be grown on Cu or Cu-Ni foils with grain sizes up to the centimeter scale^16, 17^, and recent advances in graphene growth show that even in cold wall CVD reactors it is possible to fabricate high quality monolayers of graphene^18^. However, the graphene still needs to be transferred to device-compatible substrates and the transfer process can introduce defects and consequently a low yield in functional devices^19, 20^.

In our previous work we demonstrated efficient transfer of graphene, which requires only 4 cm^2^ of the graphene-on-copper for fabrication of one 4-inch wafer with 52 devices per wafer^21^. This high-throughput transfer and large scale fabrication approach, combined with the cm-scale sizes of graphene domains^17^, will result in more reproducible performance of the GFETs.

In this work, we present a large scale fabrication of GFETs aimed for bioelectronics applications. Fabricated on a 4-inch scale, the process can be further adjusted to 6- and 8-inch processes with similar yield. Altogether, we evaluate the performance of the solution-gated GFETs (contact resistance, mobility and transconductance) depending on: (a) processing parameters, including substrate type (SiO_2_, HfO_2_, polyimide), passivation, geometric considerations and graphene channel size; (b) measurement conditions, including ionic strength of the gating solution used and applied potentials. Bio-experiments, consisting of *ex vivo* (heart tissue) and *in vitro* (HL-1 cell line and cortical neurons) recordings, prove the applicability of such graphene transistors for bioelectronics. A new passivation type, the feedline follower is introduced and argued to be better for neuronal interfacing. An electrochemical, gate leakage current induced cleaning of graphene and consecutive improvement of the GFETs performance is also investigated in the scope of this work.

## Results

**Figure 1:**
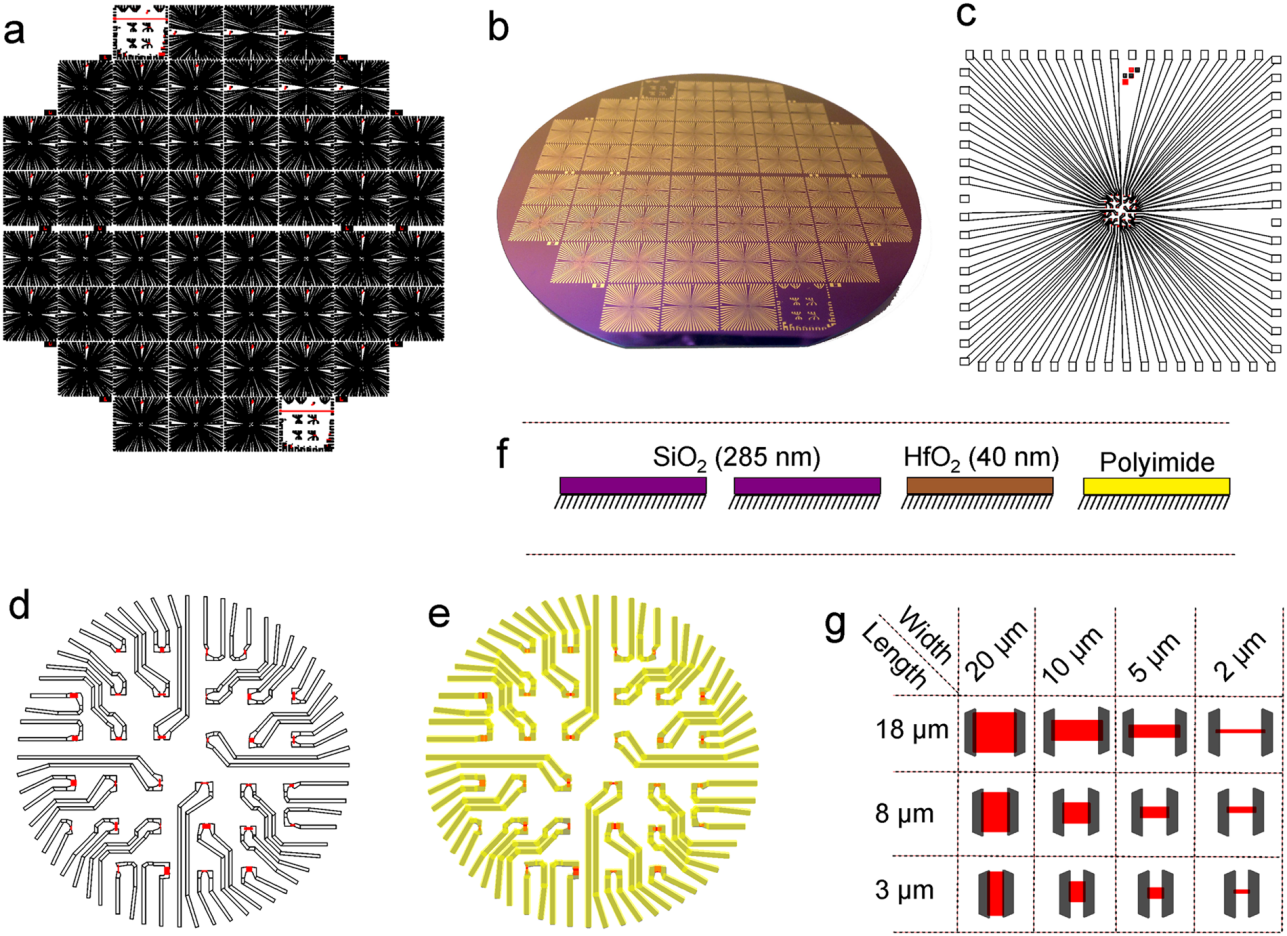
Overview of the fabrication process. (**a**) Mask design for the GFET fabrication of 52 chips on a 4-inch wafer. (**b**) Image of a fabricated wafer. Zoom into the design layout of one of the chips (**c**) with an array of 32 GFETs in the middle area (**d**). (**e**) In yellow are the parts that are covered with HD-8820 – polyimide passivation of the feedlines. (**f**) Four different substrates were analysed in this work. (**g**) The sketch shows the 12 variations in width and length of the GFETs studied in this work. Graphene is shown in red, the edges of the passivation and location of the metal feedlines are shown in grey.

In order to provide a comprehensive statistical analysis and study extensive cellular recordings, we fabricate our devices on 4-inch wafers (see Fig. 1a–b). Each wafer consists of 52 chips with different layouts. The chips (see Fig. 1c) are designed and fabricated in order to measure and track the propagation of extracellular electrical signals through the cellular layer. Therefore, it is important to have a structured array of 32 devices (6 by 6 excluding four corners) with an inter-device pitch of 200 µm (see Fig. 1d). Such devices can be fabricated on any silicon technology compatible wafer. In the manuscript we present the experimental data collected from four wafers: two SiO_2_/Si (further denoted as Si-I and Si-II), one HfO_2_/Si (Hf) and one polyimide/Si (PI) (see Fig. 1f). The Si-I, Hf and PI wafers were fabricated in a top contact method, while the Si-II wafer was fabricated with graphene metallised from two sides. As stated above, the 52 chips are fabricated with different layouts in order to fulfill diverse goals. In order to investigate the influence of the graphene area’s shape on the final performance of the device, eight chips per wafer were fabricated with varied W/L ratios of graphene. As schematically shown in Fig. 1g, there are four widths (2 µm, 5 µm, 10 µm and 20 µm) and three lengths (3 µm, 8 µm and 18 µm) of the graphene channels. However, the lengths of the channels are defined by the polyimide passivation opening (HD-8820, see Methods for details), which is not a very photostructurable resist, resulting in some variation of the final length (±0.5 µm). The accurate length of each analysed device was measured via optical microscopy. In order to provide a better interface between the GFETs and neuronal cells, we designed a new type of passivation, namely the so-called ‘feedline follower’. As schematically shown in Fig. 1e, the passivation (3 µm thick polyimide) covers only the area over the metallic feedlines.

**Figure 2:**
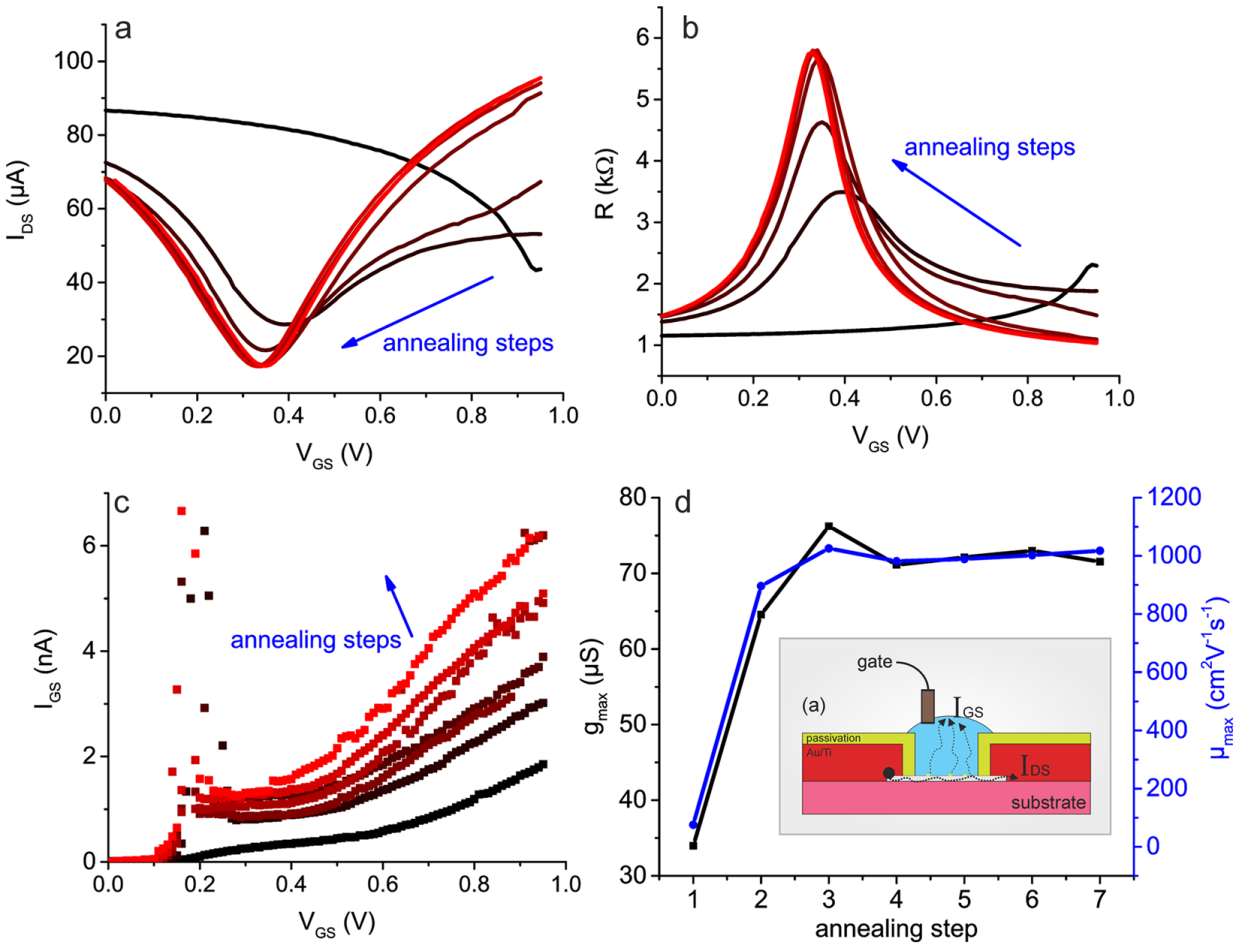
The evolution of the I_DS_-V_GS_ (**a**) and R_GS_-V_GS_ (**b**) curves over consecutive annealing steps. (**c**) The dependency of the gate leakage current on the V_GS_. (**d**) The evolution of the GFET transconductance and mobility over the annealing cycles. The inset gives the proposed mechanism of current-induced annealing of the graphene sheet under liquid physiological conditions in schematic form.

The electrical characterisation of the GFETs was performed using a Keithley 4200 semiconductor characterisation system. Drain and source electrodes of a GFET were contacted with tungsten needles and the gate was contacted via an Ag/AgCl pellet electrode inserted into an ionic solution, in our case phosphate buffered saline (PBS). The characterisation of a GFET consists of two steps: current annealing and measurement. The annealing step, was discovered during initial measurements, and is required to bring the transistor into its operative state. Failure to perform this step prevents further accurate analysis of the devices. During the annealing step, the drain-source current, I_DS_, is recorded, while the gate potential, V_GS_, is swept against the Ag/AgCl pellet electrode from 0 V to 1 V. Initial characterisation measurements of the GFETs usually indicate the charge neutrality point (e.g. Dirac point) is far within the p-doping regime (see Fig. 2a). However, every consecutive measurement brings the Dirac point to the left, until a stable position (V_Dirac_~350 mV) is reached (see Fig. 2a–b). Once annealed, further I-V sweeps do not shift the Dirac point, nor change the channel’s resistivity, indicating a clean surface of our graphene. Usually, the larger the V_GS_ sweep window is, the faster the curve stabilises. Previous investigations have reported a similar phenomenon based on current annealing, which is based on the atomic re-structuring of the graphene lattice^22–24^. However, it is unlikely that this effect is responsible for the observation in our case, considering the current/potential levels are too low to result in such an effect. The drain-source current density^22, 23^ results is on the order of 10^6^ A·cm^−2^, which provides a current density, insufficient to anneal the graphene lattice, especially considering the liquid environment. Moreover, when reducing the drain-source potential to 1 mV the effect remains, although the actual channel current density is decreased to the order of 10^4^ A·cm^−2^.

This is an interesting phenomenon, which, to the best of our knowledge, has not been previously reported in literature. We suggest that the effect originates from the removal of the cleanroom process contaminants (which dope the graphene into the p-regime) via an electrochemical reaction driven by the out-of-plane leakage current. Application of high V_GS_ potentials (up to 1.4 V *vs* a Ag/AgCl electrode, gold feedlines passivated) increases the gate leakage current up to tens of nA (see Fig. 2c). We observed that the gate leakage current I_GS_ increases with the gate source voltage V_GS_. This effect becomes more pronounced after each annealing step (see Fig. 2c). However, the gate leakage is still small compared to the drain-source current.

The removal of contaminants can result in an intermediate step, when a local doping creates a second conduction minima (pseudo Dirac point, see Supplementary Fig. S2a). Every consecutive measurement reduces the second conduction minima, finally resulting in a graphene transistor transfer curve with just one charge neutrality point (see Supplementary Fig. S2c). Evolution of the transconductance and mobility of the devices over the annealing cycles is given in the Fig. 2d. The data show that the first transfer curve is not an appropriate indicator for further data processing, but the system stabilises after the third measurement.

Following the annealing steps, the GFETs are characterised (V_GS_ sweeping 0 V – 0.8 V; V_DS_ = 0.1 V) and analysed for their performance, including transconductance, mobility, Dirac resistance, contact resistance and sheet resistance (see Supplementary Figs 3–4for details). The first two parameters (g_m_ – maximum value of transconductance and µ_max_ – maximum value of field effect mobility) are usually taken into consideration for comparison with other materials and works^25^. Remarkably, some of the values, like sheet resistance, R_S_, can be used for the rough evaluation of the GFET’s quality, while values of mobility have to be carefully examined in order to provide a correct and valuable comparison (see Supplementary Information for details).

**Figure 3:**
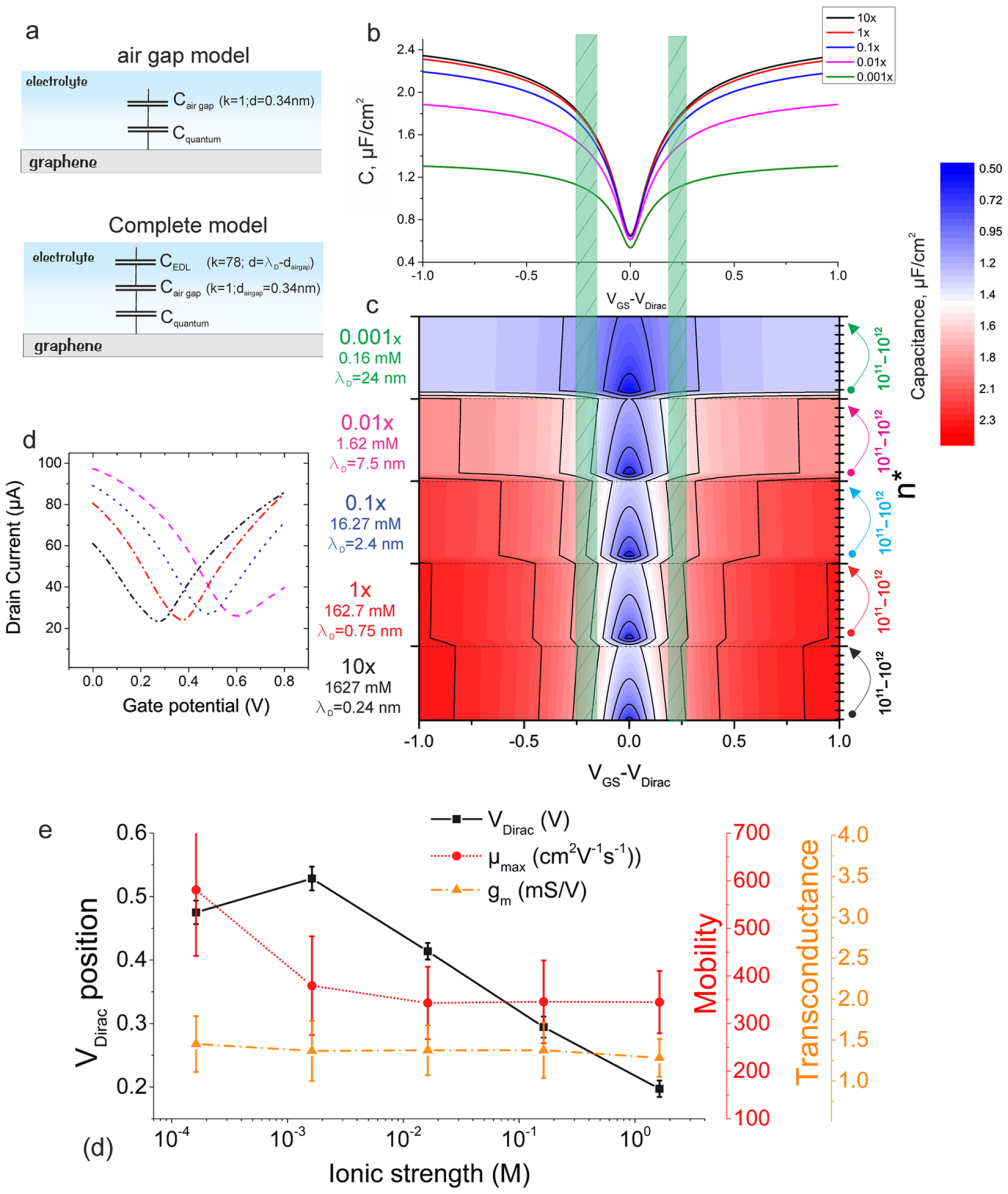
(**a**) Two capacitance models for a liquid gated GFET. (**b**) The C_total_-V_GS_ plots derived from the corresponding models, n* = 1 × 10^11^ cm^−2^. Calculations are given in Supplementary Information. (**c**) The more detailed colour plot of capacitance over V_GS_, while varying both ionic strength of the solution during measurements and n* during modeling. The green dashed areas represent the areas of maximum transconductance/mobility. (**d**) Shift of the I-V curves (position of the Dirac point) when changing the ionic strength of the solution, representing the 60 mV/decade shift due to Cl^−^ ion concentration and the change in chemical potential. In (**e**) the evolution of the three main GFET parameters are given: position of the Dirac point, mobility and transconductance over the ionic strength of the solution. Each data point is calculated from five transistors and the error bars represent the differences in their performance.

From the equation for a transistor in a linear regime^26^:

$${I}_{DS}=\frac{W}{L}\cdot {C}_{ox}\cdot \mu \cdot ({V}_{GS}-{V}_{Dirac})\cdot {V}_{DS}$$

we can calculate the mobility, $\mu =\frac{L}{W}\cdot \frac{g}{{C}_{ox}{V}_{DS}}$and the transconductance, $g=\frac{d{I}_{DS}}{d{V}_{GS}}$. Transconductance is simply a first order derivative from the transfer curve and is the most important and direct value to evaluate the sensitivity of the transistor for potentiometric biosensor applications utilizing a graphene transistor’s channel conductivity as the sensing element. Extraction of the value of the field-effect mobility, on the other hand, is a more complex value, which requires knowledge of the interface capacitance. Specifically, it is known that graphene devices, biased via a liquid gate, undergo a very complicated gating procedure. For a proper model one has to consider the electric double layer (EDL) capacitance, C_EDL_, and the quantum capacitance, C_Q_, of the graphene itself (see Fig. 3a)^27–29^. While the C_EDL_ can usually be approximated by a parallel-plate capacitor, the C_Q_ is an intrinsic property of graphene and depends on the charge carrier concentration,$\,n={(\frac{e{V}_{GS}}{\hslash {v}_{F}\sqrt{\pi }})}^{2}$, induced by the gate potential, and n*, induced by charge impurities. The concentration of induced charge impurities^28^, n*, is known to vary from 1 × 10^11^ to 1 × 10^12^ cm^−2^. An extra term, the so called air gap capacitance, C_airgap_, was later proposed to be included in the model for more precise calculations^30^. C_airgap_ is valid for hydrophobic materials (such as graphene) and high ionic concentrations of the solution gate, when the usual approach would end up in an unrealistic case of ions coming too close to the graphene surface^2, 30, 31^. Finally, we combine all three capacitances, with the calculation details given in the Supplementary Information. In order to provide a comprehensive analysis, we measured six GFETs in a PBS titration series PBS from 10x down to 0.001x. 10x PBS is a solution has an ionic strength of 1.62 M, which would theoretically result in a Debye length of 0.24 nm, however, such values are not physically possible^31^. Therefore, for the highly concentrated salt solutions, the air-gap simplified model is used for the capacitance calculations. In the model, the diffuse layer breaks down for high concentrations and steric repulsion has to be considered. Nevertheless, the overall capacitance is dominated by the air gap (which takes the place of the Stern layer in our model)^31^. Therefore we use the simpler model, taking into account only the quantum and air gap capacitances (see Fig. 3a top panel). For PBS concentrations with a lower ionic strength we use a more complicated model that additionally takes into account the capacitance of the diffuse double layer (see Fig. 3a bottom panel). Since the quantum capacitance of the graphene depends on the number of charge carriers induced by the gate potential, the overall capacitance of the liquid-gated graphene transistor depends on the V_GS_ potential. The overall C_total_ versus V_GS_ plot for different PBS concentrations is given in Fig. 3b. Calculation details are given in the Supplementary Information. The plots in Fig. 3b are calculated using n* = 1 × 10^11^ cm^−2^. In order to understand the significance of n* we have plotted the calculated capacitance over 5 values of PBS concentration while changing the assumed n* from 1 × 10^11^ cm^−2^ to 1 × 10^12^ cm^−2^ (see Fig. 3c and Supplementary Fig. S5 for a more detailed plot). The resulting behaviour shows that increasing n* increases the overall capacitance value. The changes in C_total_ over n* are the highest close to the charge neutrality point, but can be neglected in the regions of maximum transconductance/mobility (see green dashed areas in Fig. 3b–c; also see Supplementary Table S1 for details).

Transfer curves from one of the GFETs are given in Fig. 3d. The Dirac point position of the GFET shifts depending on the ionic strength of the solution used as the gate, as visible in Fig. 3d. This dependency can be explained by the change of the solution’s chemical potential when changing its molarity/ionic strength. The Dirac point shift is −83 ± 17 mV/decade, from which the 60 mV/decade of the response due to changes in the chloride concentration when diluting the PBS buffers must be subtracted. The Dirac point shift of −23 ± 17 mV per decade is small compared to that previously reported^14^, suggesting an advantageously cleaner surface^32^.

In Fig. 3e the summary of the GFET’s performance in different PBS solutions is given; while the Dirac points shift due to change in chemical potential, the transconductance and mobility values are not significantly different.

**Figure 4:**
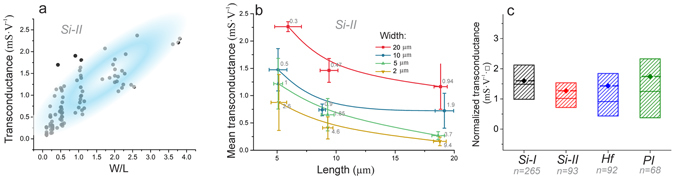
(**a**) The scatter plot (gray points) of the transconductance values for 93 devices from wafer Si-II and a blue oval showing a general linear trend. (**b**) The mean and SD values of the GFET transconductances on wafer Si-II, and their dependency on length while keeping the width constant. Numbers near each data point represent the number of squares. (**c**) The linear fitted curves of the four wafers; black: Si-I, red: Si-II, blue: Hf, green: PI. (**d**) The statistical distribution of the normalised transconductance of the four wafers; black: Si-I, red: Si-II, blue: Hf, green: PI. The box is 25% to 75%, the dashed line with diamond shows mean value and the solid line the 50% value.

Further analysis of a device’s performance is done regarding the processing parameters, i.e. substrate, width and length of the graphene area, and the type of contacts. For this purpose, over 500 GFETs from four different wafers (Si-I, Si-II, Hf, PI) with different lengths and widths were measured and analysed. The ionic solution and V_DS_ potential were kept constant for every measurement. The outcome is that the transconductance is linearly dependent on the width-to length (W/L) ratio of the device, as previously reported^33^. In Fig. 4a the values of transconductance over the W/L ratio of the graphene channel are plotted from devices fabricated on one wafer, Si-II. The fitted curve shows a linear trend (with a correlation coefficient of around 0.99). In order to show more detailed width and length dependency, we averaged the transconductance values for each width-to-length combination. The result can be seen in Fig. 4b, reproducing the above-described trend. We therefore will use the proposed normalisation of the transconductance to a number of squares (one square (□) is when W/L = 1, see Supplementary Fig. S1)^33^. Such normalisation brings more comparable values important to describe and compare intrinsic properties of GFETs. The other wafers were analysed in a similar way and the averaged fitting curves, which show the slope of the transconductance are given in Supplementary Fig. S9. From these dependencies we can extract the normalised values of transconductance, denoted further in mS·V^−1^·□, and plot in statistical details in Fig. 4c. Interestingly, in our case the mean values of the normalised transconductance do not depend strongly on the underlying substrate; however, minor differences are visible. The mean values of transconductance, normalised to V_DS_ and number of squares (□), for all five wafers are in the range of 1-2 mS·V^−1^·□.

**Figure 5:**
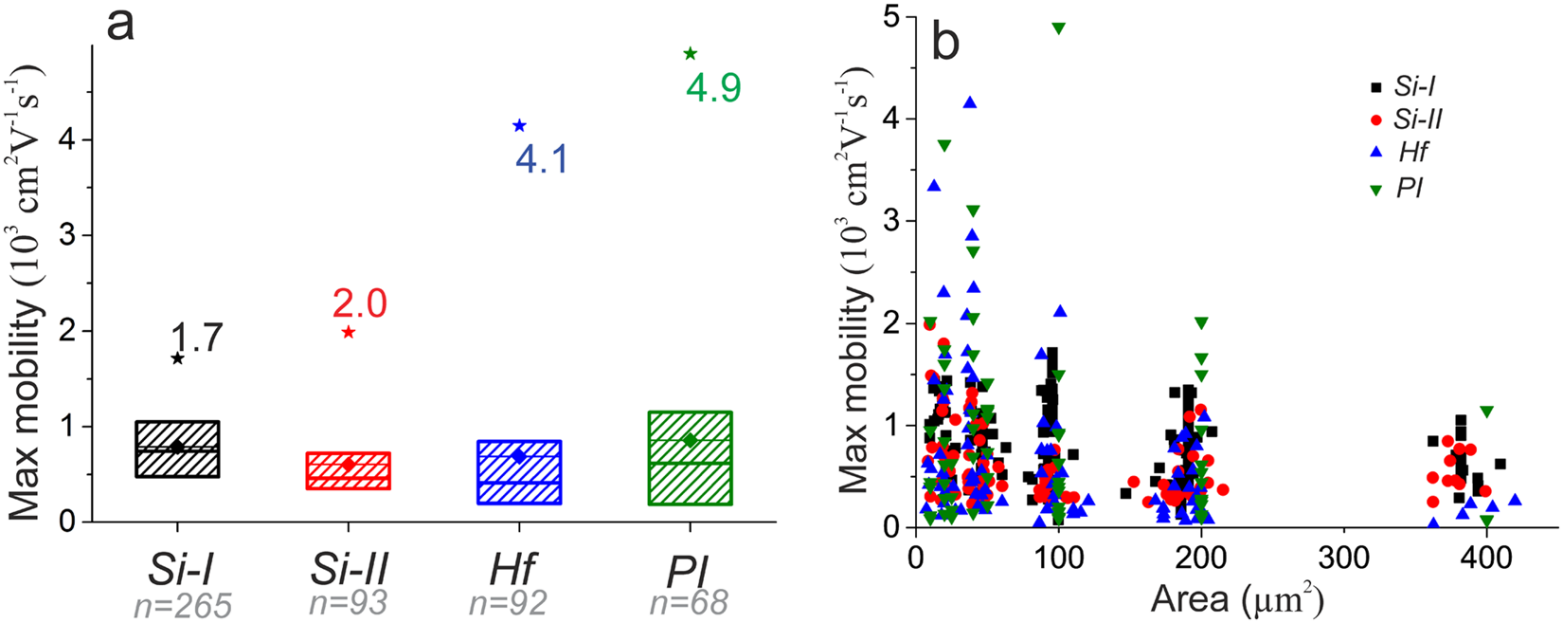
(**a**) The statistical distribution of the mobility values and their max values of the four wafers; black: Si-I, red: Si-II, blue: Hf, green: PI.. The box is 25% to 75%, the line with diamond shows mean value and the solid line the 50% value. (**b**) The scatter plot of all mobility values over the tested areas of graphene. One can see a general area-irrelevance, except for the smallest devices.

In order to understand the field effect mobility values (measured and calculated with an ionic strength of 162.7 mM), the values are averaged for each wafer (see Fig. 5a). Since the distribution of the data is not Gaussian, but skewed towards lower values, the detailed histogram plots are given in Supplementary Fig. S8. The same CVD grown graphene was used for all four wafers, and the mobility values are quite similar for all wafers (in the range of 500–1000 cm^2^·V^−1^·s^−1^). However, one can see that there are rare occurrences of extra-large mobility values especially for the HfO_2_ and polyimide substrates (Fig. 5a). In order to understand the origin of such large mobilities, we plot the data for each transistor from each wafer over the area of the graphene channel (Fig. 5b). From the Fig. 5b it becomes clear that the extremely high mobility values only happen in devices with very small channel areas (below 100 µm^2^). One of the possible explanations of this phenomena is a finite crystallinity of the graphene. Regardless of the grain sizes of the CVD grown graphene, in smaller devices there are fewer grain boundaries, reaching a situation when a GFET consists of a single graphene crystal. In this case we see the drastic increase in that transistor’s charge carrier mobility. While for the GFETs with channels above 100 μm^2^ in area, the chance of grain boundaries increases, limiting electrical performance of the GFETs^34, 35^. The principle was also shown when boundaries were specifically introduced for ion channel sensing applications^36^. Another explanation for the observed mobility values is substrate-induced scattering, which constricts the electrical properties of even single crystalline devices, as visible on the example of SiO_2_-based devices. Although the devices on SiO_2_ substrates have the largest average mobility (750 ± 350 cm^2^·V^−1^·s^−1^), the SiO_2_ substrate results in a suppression of the performance^37^ compared to HfO_2_ and polyimide substrates, where the value can reach up to 4.9 × 10^3^ cm^2^·V^−1^·s^−1^.

The last two important parameters for characterisation of the GFETs are the contact resistance, R_C_, and transfer length, L_T_. The transmission line measurement technique (TLMT) has been used to determine the parameters^38^. Details of the calculations are given in the Supplementary Information. We analysed the difference in the contact resistance for wafer Si-I (top contacted) and Si-II (double contacted) and the results can be seen in Supplementary Fig. S7. The mean value of both wafers’ sheet resistances are: R_S, Si-I_ = 1550 ± 820 Ω and R_S, Si-II_ = 1210 ± 1040 Ω. Clearly double side contacting reduces the contact resistance and therefore the overall sheet resistance of the GFETs. The computed transfer length for the wafers are: L_T, Si-I_ = 8.5 ± 2.2 µm and L_T, Si-II_ = 3.6 ± 2.2 µm. The transfer length is the path a charge carrier has to travel underneath the contact area before transfer to the feedline. Decreasing transfer length is important for further miniaturisation of the devices. This shows that the transfer length could be reduced by more than a factor of 2 using double contacted graphene.

Table 1 is provided in order to directly compare the data from previously published works^5, 8, 14, 30, 33, 39–41^ when the geometrical and environmental factors are taken into account. Interesting to see is that first of all just voltage normalised transconductance values can be misleading, and the values are extremely over/underestimated. When the values are further normalised per number of squares, the final performance shows a similar trend across publications.

**Table 1:** An overview of liquid gated graphene transistors used in literature.

	Graphene	Substrate	Ionic solution	Ionic strength, mM	W, µm	L, µm	W/L	g_m,_ *µS*	g_m_, *µS·V* ^−*1*^	g_m_, *μS·V* ^−*1*^ *·□*	µ_max_, × 10^3^ *cm* ^*2*^ *·V* ^−*1*^ *·s* ^−*1*^	V_DS_, mV
Vieira *et al*.^14^	CVD	SiO_2_	PBS	N/A	75	6.25	12	1.25	6250	520	0.75	0.2
					75	12.5	6	0.75	3750	625	1.1	0.2
					75	25	3	0.5	2500	833	1.8	0.2
Cheng *et al*.^8^	CVD	PI	PBS	N/A	60	40	1.5	512	2560	1705	N/A	200
Hess *et al*.^5^	CVD	Sapphire	PBS + NaCl	50	40	20	2	420	4200	2100	1.7	100
Dankerl *et al*.^30^	Epitaxial	SiC	PBS	N/A	40	20	2	46	460	230	0.4	100
Ohno *et al*.^39^	Exfoliated	SiO_2_	KHP	10	< 10	< 10	< 2	36	N/A	> 180^a)^	N/A	N/A
Blaschke *et al*.^40^	CVD	PI	PBS	5	20	10	2	400	4000	2000	N/A	100
Brown *et al*.^41^	CVD	SiO2	PBS + NaCl	10 + 100	40	40	1^c)^		4500	4500	7^c)^	N/A
Kireev *et al*.^33^	CVD	PIonS	PBS	162.7	20	3	6.66	1100	11000	1650	1.75^d)^	100
This work	CVD	SiO_2_	PBS	From 0.16 to 1627	2–5–10–20	3–8–18	*Var*.	*Var*.	*Var*.	1600^b)^	0.8^b)^ (2.0)^d)^	100
		HfO_2_								1430^b)^	0.7^b)^ (4.1)^d)^	
		PI								1340^b)^	0.7^b)^ (4.5)^d)^	

^a)^Calculated with the assumption V_DS_ = 100 mV; ^b)^given the mean values only; ^c)^Hall bar structure; ^d)^given the maximum value recorded for one of the devices.

**Figure 6:**
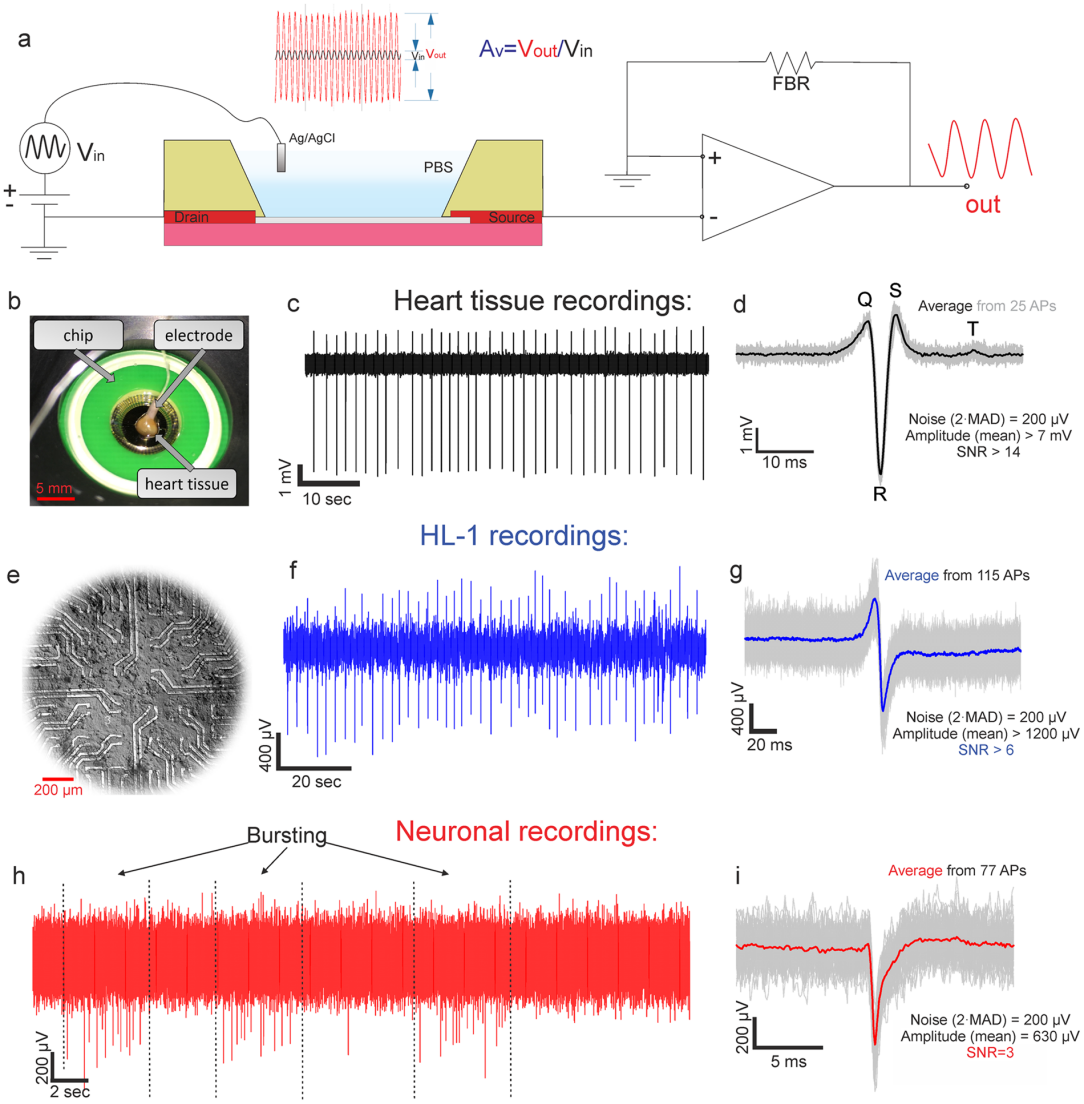
(**a**) Simplified schematics of the multichannel measurement set-up. Not to scale. (**b**) A photograph of the heart tissue on top of the chip’s surface while recording. (**c**) The time trace from one of the GFETs showing large (up to 8 mV amplitude) and frequent (40 bpm) APs. (**d**) The AP, averaged from 25 consecutive spikes with visible Q, R, S and T modes. (**e**) An optical micrograph of an HL-1 cell culture on top of a GFET chip. (**f**) A typical time trace recording of HL-1 activity recorded with a graphene transistor. (**g**) The averaged HL-1 spikes (blue) from 115 individual consecutive spikes from the chip. (**h**) The neuronal recording time trace with the inherent neuronal feature of bursting, when the neurons exhibit alternating periods of high frequency (bursts) and low frequency, intermittent, spiking. (**i**) The averaged AP (red) from 77 individual APs (gray) from the neuronal time series.

In order to show the applicability of the devices for bioelectronics applications, the devices were encapsulated and prepared for cell culture (see Supplementary Information). The devices were later measured at the custom-built multi-channel measurement set-up, the principle of which is schematically shown in Fig. 6a. The measurement routine consists of two steps. Firstly, calibration is done by applying a sine signal with a certain amplitude, V_in_, to the gate electrode, while source-drain signal amplitude, V_out_, is monitored and the overall amplification factor ${A}_{V}={V}_{out}/{V}_{in}$ is calculated. Next, in the recording/time series step, the V_DS_ is simply measured, and the changes (∆V_DS_) are transformed into ∆V_GS_ by dividing by A_V_ (see Fig. 6a). Every further presented time series data shows the actual gate potential fluctuations (∆V_GS_).

Afterwards, an embryonic heart tissue is placed on the chip’s surface (see Fig. 6b). A Ag/AgCl pellet electrode is placed as close to the tissue as possible in order to provide a stable potential through a small amount of ionic solution (supplementary medium). The time trace recording from one of the channels (GFETs) is presented in Fig. 6c showing repetitive spikes up to 7 mV with a beat rate of 30–40 beats per minute (bpm). When the action potentials (APs) from one channel are extracted and averaged, they result in a very distinct shape with visible Q, R, S and slight T regions of the electrocardiogram (Fig. 6d). The heart tissue measurements show an excellent applicability of the GFETs for *ex vivo* bioelectronics. The overall signal-to-noise ratio (SNR) of the measurements is up to 17, while considering the noise as twice the median absolute distribution (MAD, see Supplementary Information for discussion).

Cardiomyocyte-like cells (HL-1 cell line) are further cultured on the chips’ surface (see Fig. 6e). A typical time trace recording from a GFET with HL-1 action potentials is shown in Fig. 6f. The cells are beating (producing repetitive APs that propagate through the whole cellular layer) with a rate over 25 bpm and an amplitude of 1.2 ± 0.2 mV. Considering the 2·MAD noise level of 200 µV, the overall SNR is 6 ± 1, which is in the same sensitivity range as reported previously^40, 42^. The shape of the APs is shown in Fig. 5g, where over 115 consecutive spikes are averaged and the average AP is shown in blue. The shape of the action potential, in agreement with previous works, represents a very good sealing between the cell and the transistor^43, 44^.

Further, cortical neurons were cultured on top of the chips (details are given in Methods) with an approximate density of 1500 cells per mm^2^. The neuronal network was cultured for at least 14 days *in vitro* (DIV14) until mature, when the cultured neurons produce spontaneous APs that can propagate through the network. As expected, the extracellular neuronal APs are one or two orders of magnitude smaller than those of heart tissue^45^. Nevertheless, we were able to record the APs and even bursting activity of neuronal networks with the GFETs (Fig. 6h). More time trace recordings (including one after killing the cells) and live-dead fluorescence images can be found in the Supplementary Fig. S10. To our knowledge, such neuronal bursting activity recorded by graphene transistors is reported here for the first time. The average action potential (*n* = 77) is presented in Fig. 5i, showing the average amplitude around 630 µV. The noise, calculated as 2·MAD results in the value of 200 µV. Therefore, the resulting SNR is above 3.

Important to consider in further comparison is that in all our SNR measurements we use 2 × MAD instead of root mean square (RMS) for the noise value and only a 50 Hz filter applied to the recordings to remove the power line hum and to keep the signal as undisturbed as possible. In addition to that, there is a 3 kHz anti-aliasing low-pass filter installed in the amplifier system. However, an appropriate filter and use of the RMS value would result in the SNR estimation up to 5 for neuronal recordings, 10 for HL-1s and 25 for heart tissue (see Supplementary Information for details).

Understanding the shape and amplitude of the recorded neuronal action potentials is a complex task, considering that different parts of a neuronal network produce different kinds of potentials^46^. Moreover, the final signal shape depends on the signal transport from a cellular membrane to a transistor. Here the important factor is the so-called cell-chip coupling^47^. Since the cells are alive and dynamically growing, the coupling might be different from culture to culture and from chip to chip^42, 44, 47–49^. Nevertheless, our neuronal recordings from graphene transistors are in good accordance with previously published neuronal AP shape data^8^.

**Figure 7:**
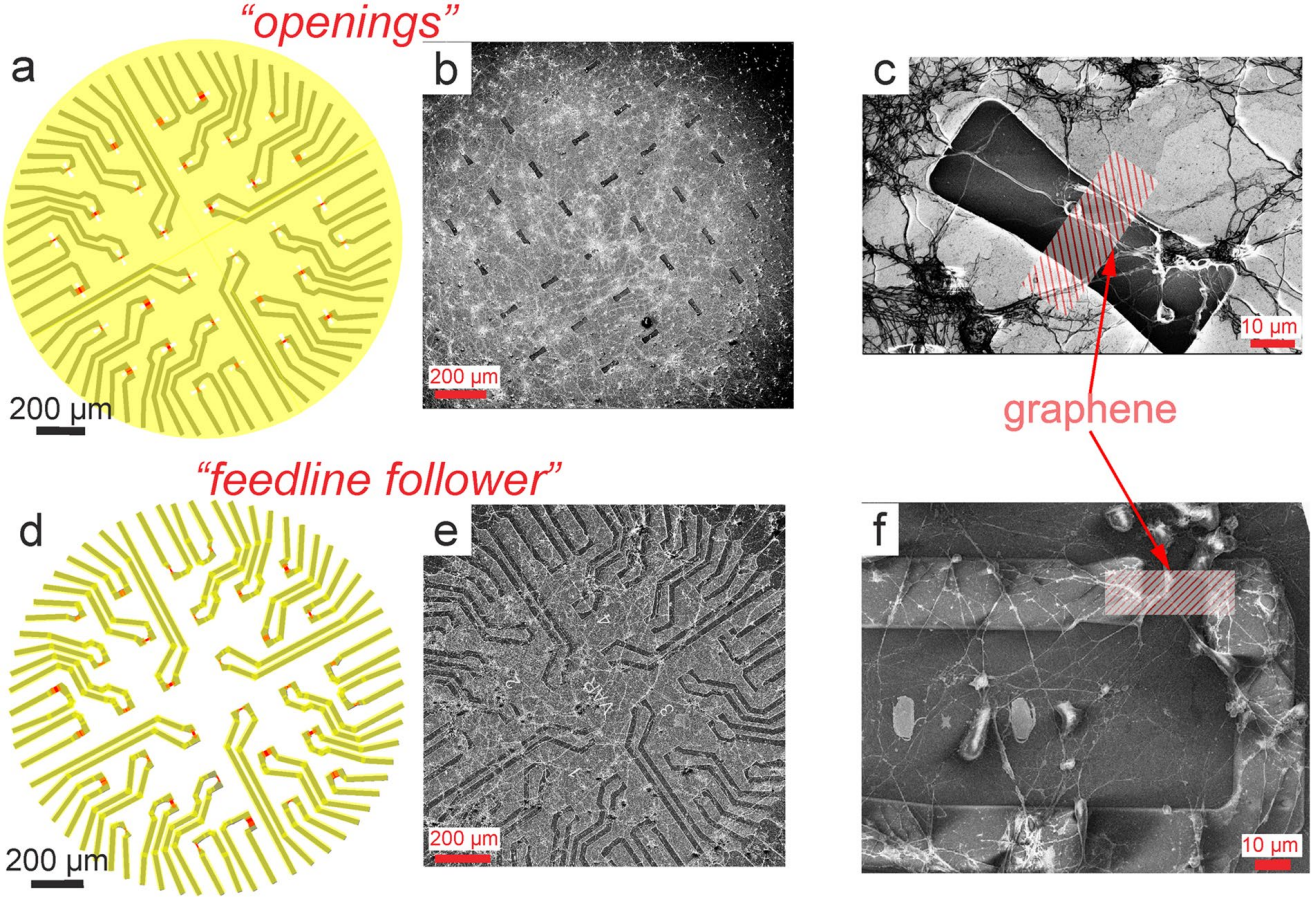
(**a**) and (**d**) represent the passivation openings and feedline follower technique respectively. The passivation is shown in yellow, openings/substrate in white and graphene in red. (**b**) and (**e**) represent SEM images of both kinds of chips with a neuronal network cultured on top, and zoom-ins into one GFET area are given in (**c**) and (**f**) for passivation openings and feedline follower technique respectively. Details on fixation and imaging are given in Methods. Due to the sputtered layer of Pt (required to visualise non-conducting neurons and dielectrics), graphene is not visible. In (**b**) and (**c**), due to a large layer of passivation, the openings look dark in the SEM image, while opposite situation in (**e**) and (**f**), where passivation covers the metal feedlines and are represented darker.

An important discussion we would like to address at the end of this work is the importance of the passivation. The most common way of passivating the transistors for bioelectronics consists of covering the whole chip’s surface except for the active area (see Fig. 7a). The openings might be useful for recording from such cells like HL-1s^50^, which form a large confluent layer all-over the surface, create a sealed environment and therefore increase the signal^51^. Neuronal networks, in contrast, do not form confluent layers, but rather grow a large network of neurites. The neurites could be as small as 1 µm in diameter. Therefore, neurons usually do not cover the entire passivation opening, and the large gap distance is compounded by unsealed areas at the cell edges (see Fig. 7b–c).

For this work we designed a new kind of passivation, the so-called feedline follower, where the passivation covers only the area over the metallic feedlines, as it was shown in Fig. 1d. We believe that using this passivation technique, helps to create a better interface between graphene gates and neurons by preventing membrane bending stresses as the cell approaches the graphene and therefore reduces the gap distance between the neuron and the gate (Fig. 7e–f).

Moreover, there is always a trade-off between low-noise recording interface and good cell-sensor coupling. Considering that the GFETs’ multichannel measurement setup exhibits large noise, we cannot afford to increase the noise by creating a seal. At the same time, graphene is a purely two-dimensional material and it is important to bring the cell body as close to the graphene’s surface as possible. We believe that such new passivation is more suitable for the graphene-based devices, since the neuronal culture then grows consistently closer to the level of the graphene gates.

## Methods

### Graphene growth and device fabrication

The graphene was CVD grown on 25 µm thick copper foils in an Ar/H_2_/CH_4_ gas mixture (see Supplementary Fig. S12 for Raman spectra). A thin layer of poly(methyl methacrylate) (PMMA) was used as a support layer during the transfer. In order to not waste the graphene layer, we used the modified, high-throughput transfer technique^21^. The graphene was transferred onto the wafer with pre-fabricated Au/Ti markers. AZ-5214 photoresist (MicroChemicals GmbH) was then spin-coated (3000 rpm), soft baked (110 °C for one minute), exposed to i-line UV light with a dose of 55 mJ/cm^2^, developed in 0.26% TMAH solution, and was used to protect the graphene active areas during exposure to oxygen plasma (300 W, 200 sccm, 5 minutes). The stack of 10 nm Ti and 90 nm Au was e-beam assisted evaporated on the wafer through a pre-defined structure of lift-off resists. For this purpose, LOR-3B (MicroChemicals GmbH) was spin-coated (3000 rpm) and soft baked (150 °C for 5 minutes) on top of another resist, nLOF-2020 (MicroChemicals GmbH), spin-coated (3000 rpm) and soft baked (100 °C for 2 minutes) in advance. The resists were exposed to i-line UV light at a dose of 40 mJ/cm^2^, post-exposure baked (110 °C for 1 minute) and developed in 0.26% TMAH. Photostructurable polyimide, HD8820 (HD Microsystems) was used in the last step to form the passivation. Spin-coated at 5000 rpm, soft-baked at 120 °C (with a slow ramp), exposed to 250 mJ/cm^2^ i-line UV light, developed in 0.26% TMAH for 80 seconds and hard baked (double slow ramp in N_2_ atmosphere: 4 °C/min up to 200 °C, 30 minutes hold; followed by 2.5 °C/min up to 350 °C, 30 minutes hold; slow cooling down to room temperature), the polyimide formed a pinhole-free, 3 µm thick passivation. Post-cleanroom treatments, such as device encapsulation protocols are given in the Supplementary Information.

### HL-1 cell culture

The cardiomyocyte-like cell line HL-1 was cultured in T25 flasks^51^. Prior to the seeding, the chips were cleaned with 70% ethanol and coated with fibronectin/gelatin solution (5 µg/mL and 0.2 mg/mL, respectively). When reaching 100% confluency, the cells were passaged and seeded on top of the GFETs at 200 cells/mm^2^. The chips were then placed in an incubator (37 °C and 5% CO_2_) for the cells to mature. Claycomb medium, supplemented with 10% fetal bovine serum, 100U/ml-100µg/ml penicillin-streptomycin, 0.1 mM norepinephrine and 2 mM L-glutamine was exchanged every day (100%) and two hours before the measurements.

### Heart tissue preparation

The heart tissue was prepared by dissecting embryonic tissue from an E18 Wistar rat. The heart of an embryo is quickly isolated, washed in Hank’s balanced salt solution (HBSS), then stored and measured in supplemented Claycomb medium (see HL-1 cell culture).

### Neuronal culture

The primary cortical neurons were isolated from E18 Wistar rats^52^. Prior to culturing the cells on a chip, a glass ring was mounted onto the chip to form a culture container and the surface of the chip was coated with GpECM (4 µg/mL Gelatin, 0.01 mg/mL poly-D-lysine and 0.0924 mg/mL extracellular matrix in Gey’s Balanced Salt Solution) for improved cellular adhesion. The cells were diluted in Neurobasal medium (Life Technologies), supplemented with B27 (1% (v/v), Gibco), L-glutamine (0.5 mM, Gibco) and Gentamicin (0.05 mg/mL), and plated on top of the chip with an approximate density of 1500 cells per mm^2^. Chips were kept in an incubator (37 °C and 5% CO2) and the supplemented Neurobasal medium was exchanged with 100% fresh supplemented medium two hours after the plating then with 50% fresh supplemented medium twice per week and one day before the measurements. The neurons were grown until at least DIV14. Measurements were performed on DIV 14–26.

### Live-dead imaging

Live-Dead staining was performed using 1 µg/ml Calcein-AM and 2 µM Ethidium Homodimer (both Life Technologies) in supplemented cell growth medium to stain live and dead cells in green and red, respectively. Cells and dyes were incubated for 15 minutes in a 37 °C incubator or on a 37 °C hot plate (if performed after the measurements). The samples were observed using an Axio Imager Z1 microscope (Carl Zeiss).

### Fixation and imaging

In order to see the topography of the neuronal network, a high-resolution image is required. We used scanning electron microscopy (SEM) to observe the outgrowth of neurons on the chip surface. It is important to use a special preparation technique in order to image biological specimens due to their normal existence in an aqueous environment and lack of conductivity^53^. After the culture, the samples are chemically fixed using a 3.2% glutaraldehyde solution in PBS for 15 minutes at room temperature. Following that dehydration is performed in a series of ethanol washing steps (from 10% to 100% of ethanol concentration). The samples are then stored in 100% ethanol until they are dried via the CO_2_ critical point drying (CPD) technique. In order to improve the charge flow, the samples were sputtered with approximately 10 nm of platinum prior to the SEM imaging. Figure 7 and Supplementary Fig. S13 show examples of the neuronal culture on top of GFET chips.

If not stated otherwise, all chemicals were purchased from Sigma Aldrich. The experiments were done with the approval of the Landesumweltamt für Natur, Umwelt und Verbraucherschutz Nordrhein-Westfalen, Recklinghausen, Germany, number 84–02.04.2015.A173.

### Data availability

The data that support the findings of this study are available from the corresponding author on request.

## Electronic supplementary material

Supplementary
