# Supplementary material for: Graphene transistors for interfacing with cells: towards a deeper understanding of liquid gating and sensitivity
Source: Sci Rep. 2017 Jul 27;7:6658. doi: 10.1038/s41598-017-06906-5 (PMC5532278; doi:10.1038/s41598-017-06906-5)
Supplement: Supplementary file 1 — Supplementary [file 41598_2017_6906_MOESM1_ESM.pdf]

Supplementary Information for:

Graphene transistors for interfacing with cells: towards a deeper understanding of liquid gating and sensitivity

*Dmitry Kireev[1], Max Brambach[1], Silke Seyock[1], Vanessa Maybeck[1], Wangyang Fu[2], Bernhard Wolfrum[1,3], Andreas Offenhäusser[1]**

[1]Institute of Bioelectronics (PGI-8/ICS-8), Forschungszentrum Jülich, 52425 Jülich, Germany

[2]Faculty of Science, Leiden Institute of Chemistry, Supramolecular & Biomaterials Chemistry, Gorlaeus Laboratories, Einsteinweg 55, 2333 CC Leiden, The Netherlands

[3]Neuroelectronics, Munich School of Bioengineering, Technical University of Munich (TUM), Germany & BCCN Munich, Boltzmannstr. 11, 85748 Garching, Germany

* E-mail: a.offenhaeusser@fz-juelich.de; Tel.: +49 2461 61-2330.

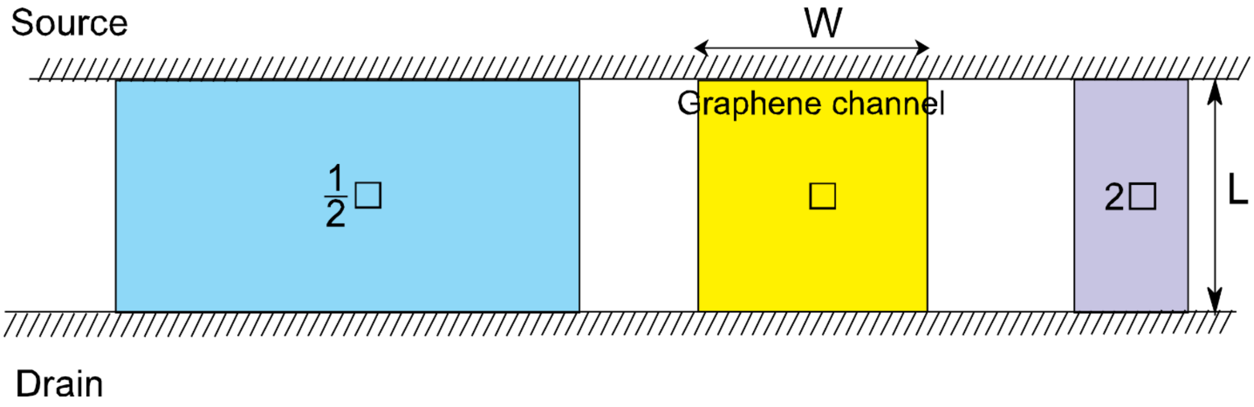

Supplementary Figure S1. A sketch for understanding the geometrical value of □ in the performance of the GFETs.

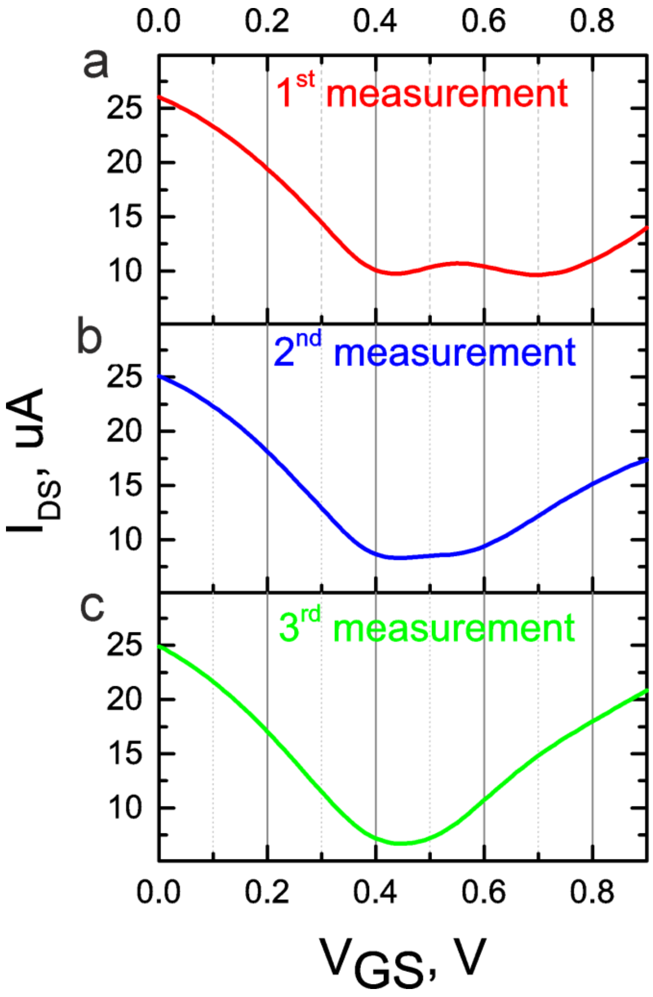

Supplementary Figure S2. The three consecutive I-V curves recorded consecutively. While the first recording (a) shows some kind of double Dirac behavior, it starts to disappear in the second recording (b) and is completely gone after the third one (c).

Data analysis flow and selection criteria

The complete data processing is depicted in the flowchart (Supplementary Fig. S3).

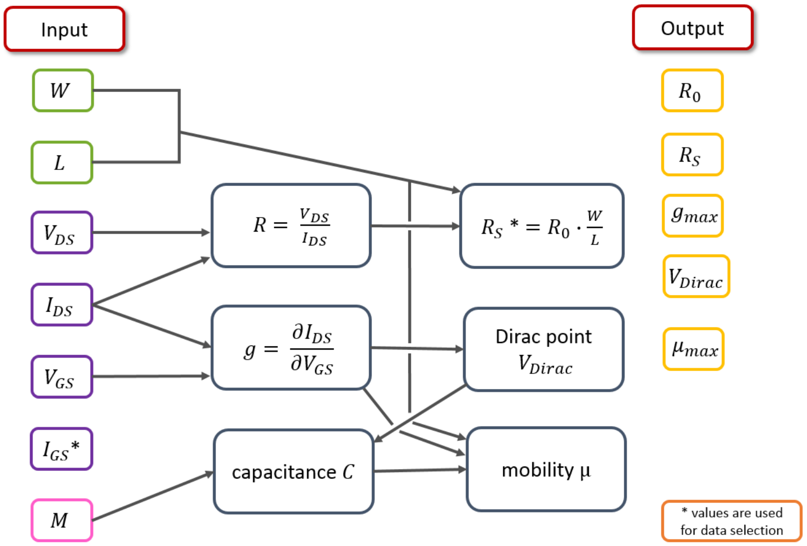

Supplementary Figure S3. A Mind diagram for data selection, analysis and calculation.

The set of recorded I-V curves went through two steps of selection before going into complete analysis:

1. Elimination of clearly damaged devices by interpreting the I-V curve shape. See Supplementary Fig. S4a for an example of a clearly damaged GFET that still shows some ambipolar behavior, but will not be considered in the following evaluation;
2. Further selection was done statistically to find out which devices behave normally, but their performance is out of range. The quantity considered for this process was the sheet resistance, or resistance per square, $Rs=R_0*L/W$. This is an inherent property of the graphene and should be equal for every GFET independent of its shape. The values of Rs are plotted in a histogram (Supplementary Fig. S4b) and fitted with a logarithmic normal distribution: $f(x) = A \cdot e^{-\frac{(Log(x)-\zeta)^2}{2\sigma^2}}$. From this fit, the mean value and the standard deviation were extracted (Supplementary Fig. S4c). For further analysis only values within ξ-σ were considered.

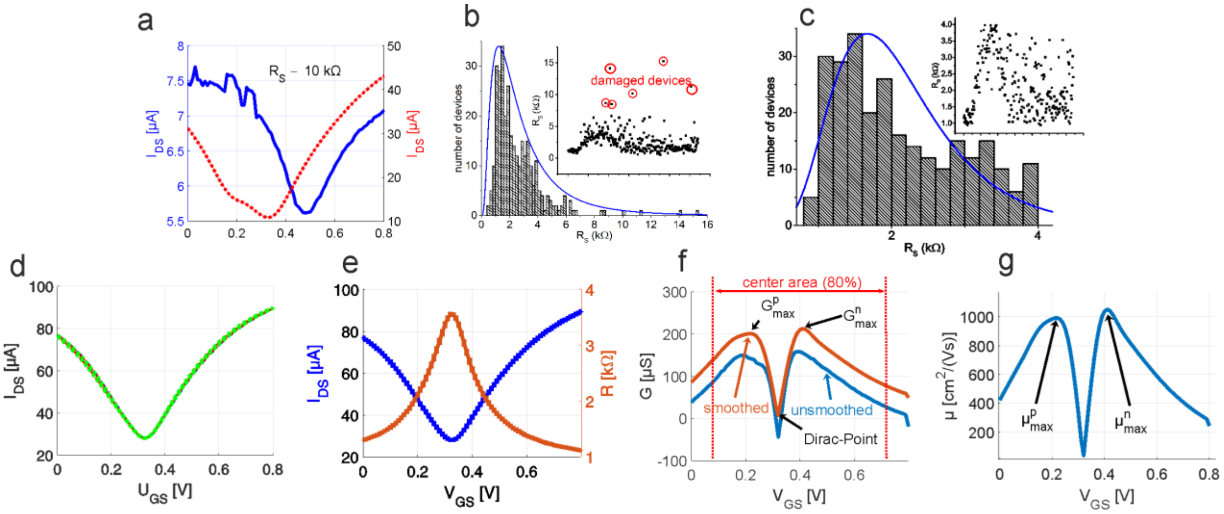

Supplementary Figure S4. (a) Two I-V curves of damaged devices, one of comparably large resistance (blue) and one of double Dirac behavior (red). (b) A statistical distribution of the Sheet resistance, Rs, values. (c) The statistical distribution after removing the damaged devices from analysis. (d) The averaged I-V curve from three measurement cycles. (e) The same I-V curve plus Resistance plot. (f) The derived transconductance plot; unsmoothed (in blue) and smoothed (in orange) to get the maximum transconductance values and their positions. Important to note the plots are shifted by 50 µS on the y-scale. (g) Mobility plot with two max values for electron and hole conductance valleys.

As soon as a device is stable, three I-V curves are recorded, then each data point is averaged and a mean characteristics are plotted (Supplementary Fig. S4d). First, the resistance is simply computed at each data point as $R = \frac{\Delta I_{DS}}{\Delta V_{DS}}$ and plotted over V_{GS} (Supplementary Fig. S4e). The characteristic quantity R_0 is the value of R_S at $V_{GS} = 0$ V. Multiplying R_0 with the ratio W/L, one gets the sheet resistance R_S that was used for the selection of the data. Furthermore, the transconductance can be computed by $g = \frac{\delta I_{DS}}{\delta V_{GS}}$. Since the data of I_{DS} is not smooth enough for numerical derivation, a Savitzki-Golay smoothing algorithm was applied to the data. The polynomial order of the algorithm was set to 3, and the number of considered data points was 21. The transconductance plot shows two peaks (see Supplementary Fig. S4f). These are the points of maximum transconductance for the hole- and electron conduction with their values g_{max}^{p} (left) and g_{max}^{n} (right) and their positions $V_{GS}(g_{\text{max}}^{p})$, and $V_{GS}(g_{\text{max}}^{n})$. The transconductance graph has a significant dip, which is then used to compute the Dirac Point position. The liquid gate capacitance C_{total} was computed as described below. Since PBS was used as the ionic solution, the n* was set to 10^{11} cm^{-2}, in accordance with the literature,[S1,S2,S3]. The PBS's molarity of 162.7 mM results in a Debye length of 0.754 nm.[S4] If a solution with a different molarity was used, λ_D was computed accordingly.The mobility was computed using the following equation:[S5] $\mu = \frac{L}{W} \cdot \frac{g}{C_{ox} V_{DS}}$.The points of maximum mobility μ_{max}^{p} and μ_{max}^{p} are determined similarly to the points of maximum transconductance (Supplementary Fig. S4g).

Computation of the interface capacitance

The applied voltage shifts the Fermi energy in the graphene area. However, if no V_{GS} is applied, the Fermi energy is still shifted due to the combined chemical potential of the ionic solution and the gate electrode. The application of a gate voltage induces the formation of an electrical double layer at the interface of the graphene and solution. The overall capacitance consists of three parts: quantum capacitance C_Q, air gap parallel plate capacitance C_{airgap}, and EDL parallel plate capacitance C_{EDL}.[S6] It is common to state the capacitance normalized to the area of the capacitor. Therefore all further C values given are capacitances per unit area.

The quantum capacitance is the same for every device and was computed using the formula:[S1] $C_Q = \frac{2e^2}{\hbar v_F \sqrt{\pi}} \cdot \sqrt{|n_G| + |n^*|},\ n_G = \left(\frac{eV_{GS}}{\hbar v_F \sqrt{\pi}}\right)^2$.

In this formula, e is the elementary charge, $\hbar$ the reduced Planck constant, $v_F \approx {}^{c}/_{300}$ the Fermi velocity (c is the speed of light), n_G and n* the carrier concentrations induced by gate potential and charged impurities, respectively. The n* is a parameter of the environment and the purity of the graphene. As explored previously, the n* value usually is between 10^{11} and 10^{12} cm^{-2}.

The air-gap capacitance was introduced recently and is computed using the standard formula $C_{airgap} = \frac{\varepsilon_r \varepsilon_0}{d}$where ε_r is the permittivity of the dielectric, ε_0 the vacuum permittivity and d the distance between the plates. In the case of the air-gap layer, $\varepsilon_r = 1$ and $d = 0.34\ nm.$ [S7] For the EDL calculation we use, $\varepsilon_r = 78$ and $d = \lambda_D - 0.34\ nm$, where λ_D is the Debye length which can be computed using the formula:[S8]

$$\lambda_D = \frac{0.304}{\sqrt{M}}\ [nm]$$

with M being the molarity of the ionic solution used.

The three capacitances are connected in series. Therefore the total capacitance C_{total} is $\frac{1}{C_{total}} = \frac{1}{C_Q} + \frac{1}{C_{airgap}} + \frac{1}{C_{EDL}}$

To model the system correctly, for the 10x PBS case, the ionic strength results in a Debye length <0.34nm, which in general indicates a non-physical estimation when ions are too close to the graphene surface. In order to overcome the problem, only C_Q and C_{airgap} parameters are taken into consideration. [S7]

Importance of the n* parameters is also investigated. Below are plots of the capacitances while varying the n* from 10^{11} cm^{-2} to 10^{12} cm^{-2}.

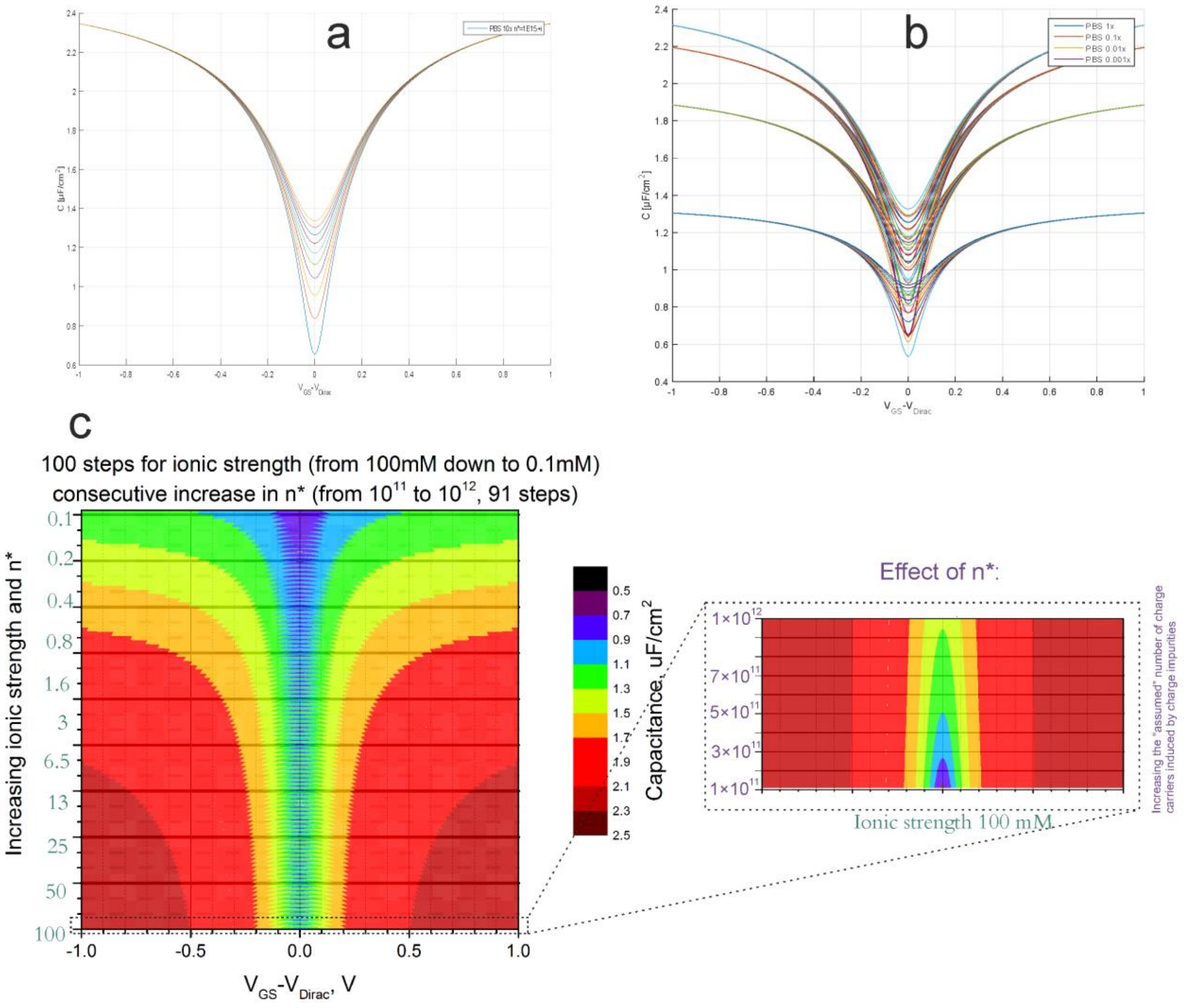

Supplementary Figure S5. (a) The capacitance plot for 10x PBS, varying n* from 1×10^{11} to 1×10^{12} cm^{-2}. (b) The capacitance plots for PBS dilutions ≤1 while varying n* from 1×10^{11} to 1×10^{12} cm^{-2} (c) The color plot for changes in capacitance with varied ionic strength and the assumed n* probed from 1×10^{11} to 1×10^{12} cm^{-2}.

Supplementary Table S1.The changes in mobility for one GFET while varying both the ionic strength and n*. The mobility is given in $cm^2 \cdot V^{-1} \cdot s^{-1}$.

**n***	**1×10^{11}**	**2×10^{11}**	**3×10^{11}**	**4×10^{11}**	**5×10^{11}**	**6×10^{11}**	**7×10^{11}**	**8×10^{11}**	**9×10^{11}**	**1×10^{12}**
**0.001x PBS**	832.89	832.70	832.51	832.33	832.14	831.96	831.78	831.60	831.42	831.24
**0.01x PBS**	575.68	575.54	575.40	575.27	575.13	575.00	574.87	574.74	574.61	574.48
**0.1x PBS**	486.78	486.53	486.29	486.04	485.80	485.56	485.32	485.08	484.85	484.61
**1x PBS**	519.32	518.70	518.09	517.49	516.90	516.32	515.75	515.18	514.63	514.08
**10x PBS**	471.80	470.44	469.12	467.84	466.60	465.38	464.20	463.05	461.93	460.84

An example of a wrong interface capacitance modeling

Here, we would like to present an example of what happens when the Cox is not modeled correctly. We show that the resulting Cox values are several orders of magnitude higher/lower than correct values. In one of the incorrect models (Supplementary Figs S6a-d) is taken into account, the resulting interface capacitance can be under-/over- estimated up to a factor of 100 (for 0.001x PBS), which will lead to over-/under- estimation of mobility.

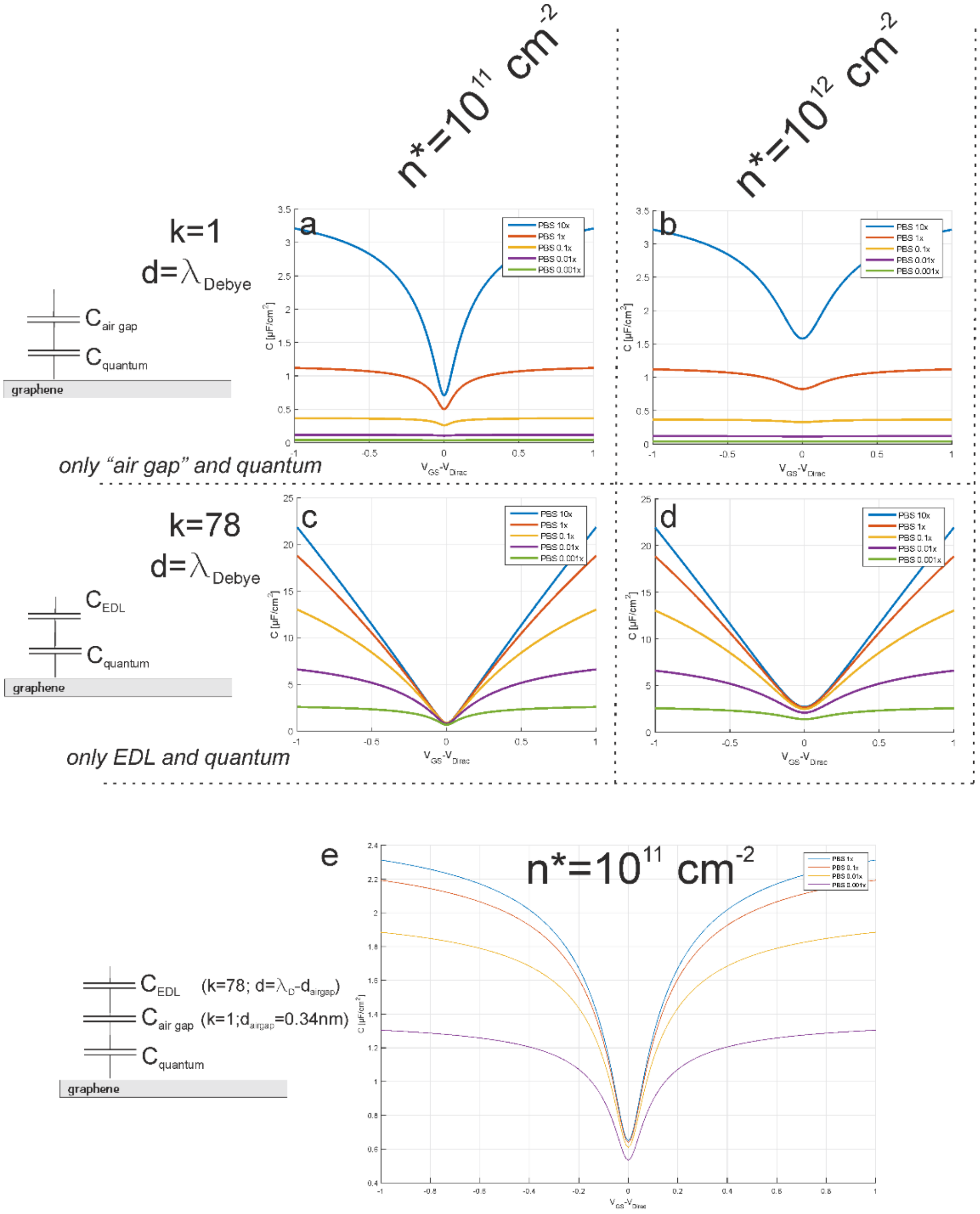

Supplementary Figure S6. (a)-(d) Four examples of incorrect interface capacitance modeling, which could be done if both EDL and air gap are not taken into consideration together. (e) The correct modeling of capacitance for 1x-0.001x PBS solutions.

Contact resistance and transfer length

The transmission line measurement technique (TLMT) has been used to determine the L_T and R_C. The resistances, R_0, of the transistors are plotted against their channel lengths, L. The data points form groups depending on the channel width W. For each group there is a linear relation between L and R_0. A linear regression with slope, ***a,*** and intercept, ***b,*** yields the following quantities: sheet resistance R_S = a*W of the graphene sheet, contact resistance R_C = b/2 and transfer length L_T = b/2a, which is the average distance that the charge carriers travel under the contacts.

The contact resistance decreases with increasing channel width (see Supplementary Fig. 7 and Supplementary Table 2). This could be expected since the larger W, the larger the overall contact area and the lower the contact resistance. R_C multiplied by W a parameter of material, and therefore, these values can be averaged for each wafer: $R_{C,\ Si\text{-}I}\cdot W = 8230 \pm 1910\ \Omega\cdot\mu m$, $R_{C,\ Si\text{-}II}\cdot W = 5300 \pm 1550\ \Omega\cdot\mu m$.

$R_C\cdot W$ is smaller for Si-II than for Si-I. This is most likely due to the double contacted graphene.

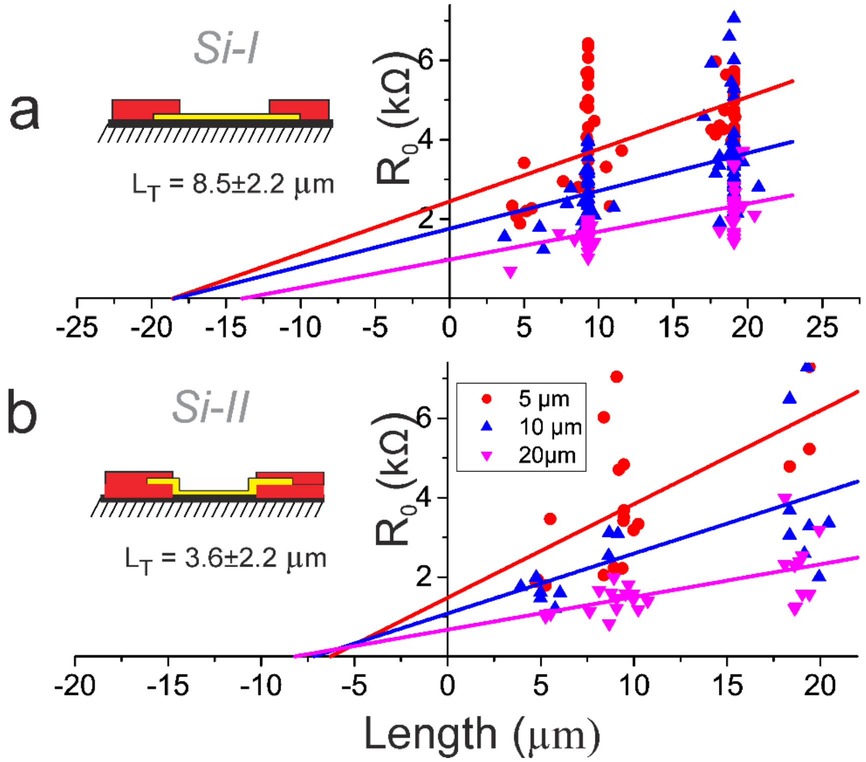

Supplementary Figure S7. The TLM plots for wafers with single (Si-I, n=265) and double (Si-II, n=93) contacted graphene.

Supplementary Table S2. Statistical analysis of the contact resistance and transfer length for Si-I and Si-II wafers

wafer	$W(\mu m)$	$a(\Omega/\mu m)$	$b(\Omega)$	n_{df}	$R_C(\Omega)$	$R_S(\Omega)$	$L_T(\mu m)$
Si1	20	70 ± 11	980 ± 150	64	490 ± 80	1420 ± 220	6.9 ± 1.5
	10	95 ± 20	1760 ± 320	106	880 ± 160	950 ± 200	9.2 ± 2.6
	5	132 ± 27	2440 ± 400	68	1220 ± 200	660 ± 140	9.3 ± 2.4
Si2	20	82 ± 22	680 ± 300	24	340 ± 150	1650 ± 440	4.1 ± 2.1
	10	157 ± 50	1080 ± 700	15	540 ± 360	1570 ± 500	3.5 ± 2.5
	5	236 ± 74	1480 ± 800	17	740 ± 400	1780 ± 370	3.1 ± 2.0

The transfer lengths, calculated for both wafers are: $L_{T,\,Si\text{-}I}$ = 8.5±2.2 um, $L_{T,\,Si\text{-}II}$ = 3.6±2.2 um.

This shows that the transfer length could be reduced by more than a factor of 2 using double contacted graphene. The TLMT implies that the sheet resistance, $R_S = (R_0 - 2R_C) \cdot \frac{W}{L}$, is an inherent property of the graphene and should therefore be constant for the devices on one wafer. If $R_C \ll R_0$ it can be neglected, leading to $R_S^* = R_0 \cdot \frac{W}{L}$. In our case, R_S^* ranges from 2 kΩ to 4 kΩ. This is more than double the value of the common sheet resistance, R_S = 1.6 kΩ, for graphene on silicon dioxide substrates.[S9] However, if the contact resistance is taken into account, the values for the sheet resistance are $R_{S,\,Si1}$ = 1010 ± 380 Ω, $R_{S,\,Si2} \cdot W$ = 1670 ± 440 Ω. Although the standard deviations are very large, the mean values of R_S are much closer to the literature value than of R_S^*. This shows that the contact resistance can not be neglected when computing the sheet resistance of a GFET.

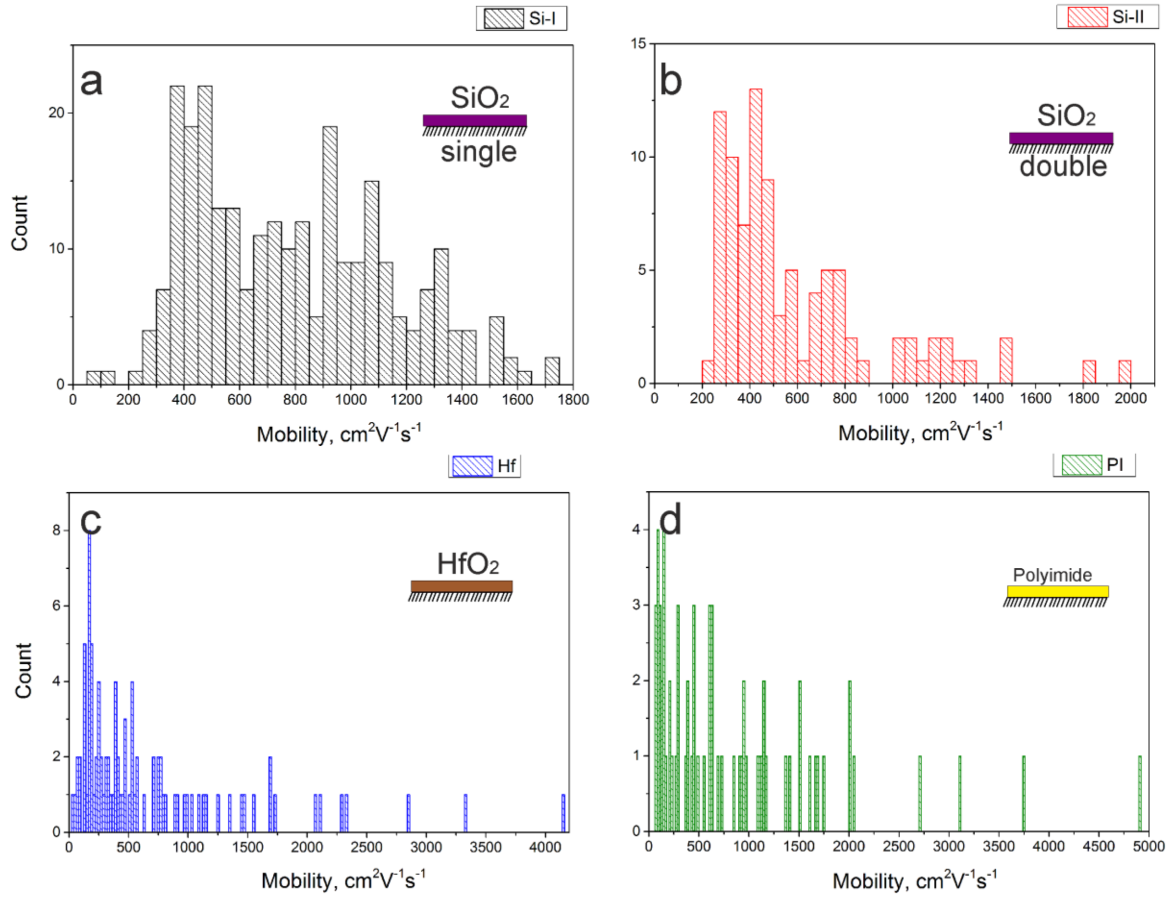

Supplementary Figure S8. Histogram distribution of mobility values for wafers Si-I (a), Si-II (b), Hf (c), and PI (d).

Details of transconductance dependency on W/L ratio

For detailed quantification of the transconductance's dependency on W/L ratio, see Supplementary Fig. S9. Then a linear regression was performed yielding coefficients *a* and *b*. The results are summarized in Supplementary Table S3. All wafers have good mean g_{max}/V_{DS} values, ranging from 0.45 mS·V^{-1} to 1.1 mS·V^{-1}.

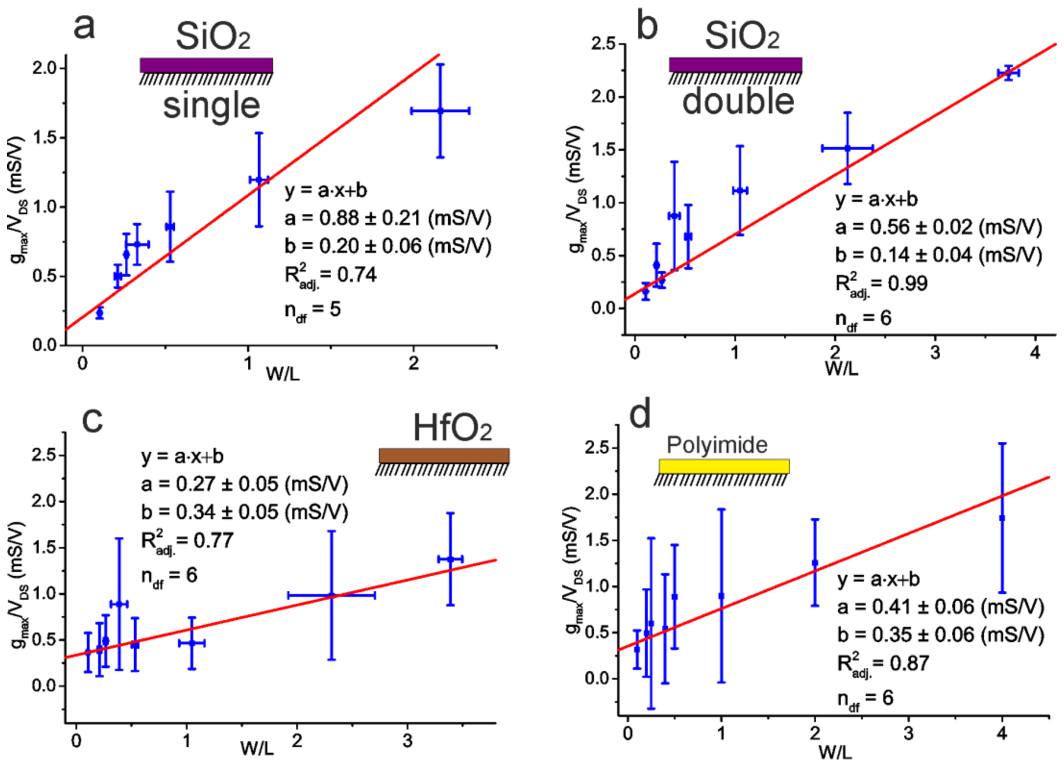

Supplementary Figure S9. Maximum transconductance, g_{max}, against the width to length ratio. The two quantities are linearly correlated.

Supplementary Table S3. The relation between maximum transconductance g_{max} and width to length ratio.

**Wafer**	**Corr. coef (%)**	**a** (mS·V^{-1})	**b** (mS·V^{-1})
Si-I	78.4	0.88±0.21	0.20±0.06
Si-II	81.0	0.56±0.02	0.14±0.14
Hf	47.2	0.27±0.05	0.34±0.05
PI	42.6	0.41±0.06	0.35±0.06

Chip encapsulation for cell culture

To use the graphene transistor arrays for cellular measurements on chips, they have to be prepared for the cell culture environment. That includes contacting the chip to a carrier with standardized contacts and the encapsulation of the chip. Encapsulation is necessary to make sure that the chip is not damaged by liquid and to provide biocompatible material for all surfaces in contact with the cell culture. For contacting the chip to the carrier the so called ’flip chip’ procedure is applied. In that procedure, the chip upward facing contacts on chip are soldered to a printed circuit board carrier with downward facing contact pads and all contacts are connected at once.

At first the carrier is tempered at 180°C using a hotplate. Then, soldering paste is applied to the inner contacts of the carrier. The chip is then placed on the carrier with the chip and carrier contacts facing each other. If the chip consists of a transparent substrate (e.g. sapphire), the alignment of the contacts can be done under the microscope. When the substrate is not transparent (e.g. silicon), the surface tension between the gold contacts and the soldering paste has to be used as an indicator of alignment. With the chip aligned, the carrier is removed from the hotplate for the soldering paste to cure. After cooling down, Epoxy (EPO-TEK- 302-3M) is applied to glue the chip to the carrier and isolate between solder points. The advantage of this procedure is that the whole chip is contacted to the carrier at once. This saves a lot of time compared to the alternative time-consuming procedure of wire bonding. The soldering part of the flip chip technique only needs five minutes. The downside of the flip chip procedure compared to bonding is the loss of working devices on the chip due to misalignment.

As mentioned above, the encapsulation is done to save the chip from being damaged by liquid and to create a cell-friendly environment. Using PDMS, two glass rings are glued on the carrier (see Fig. 5b). They are later used for holding the cell culture medium. PDMS is also applied to the area of the carrier between the rings that contacts the cell medium. This prevents toxic substances from dissolving from the carrier into the nutrient solution where it can damage the cells.

Neuronal culture

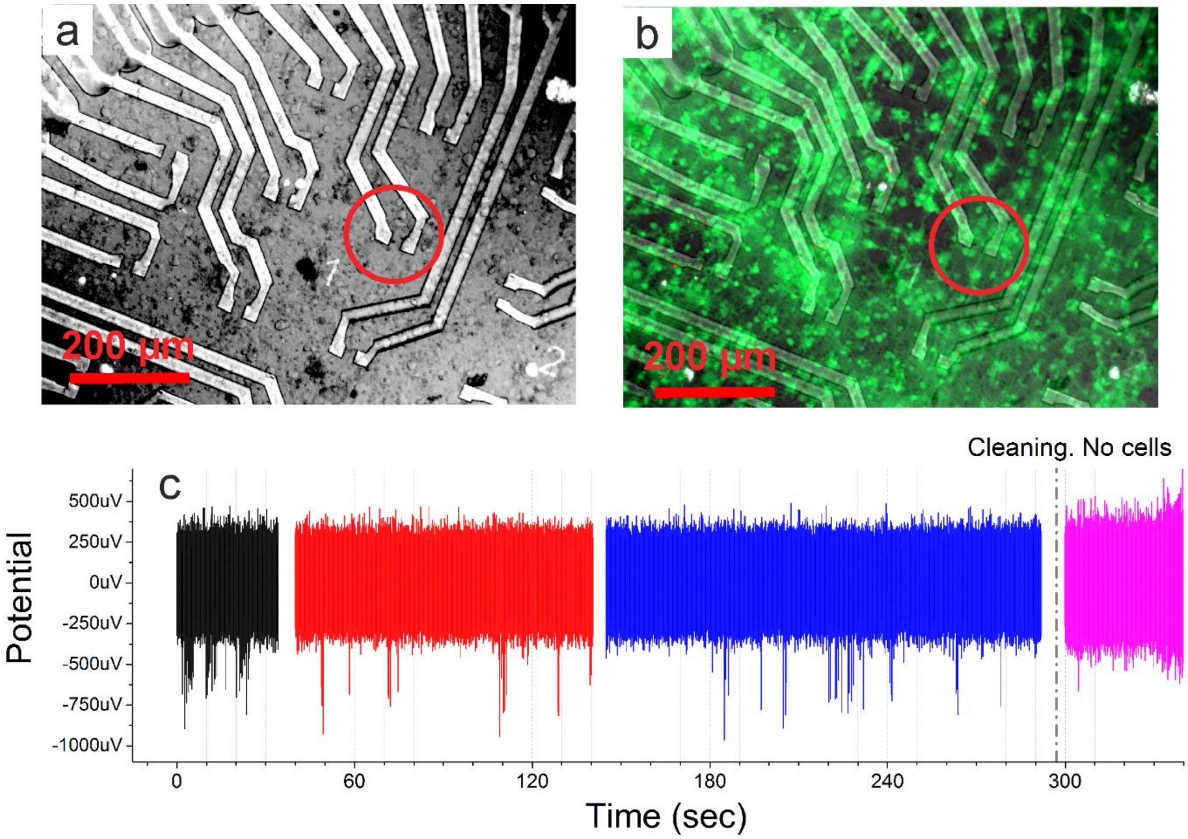

Supplementary Figure S10. (a) and (b) are the DIC and live-dead stained fluorescence image of a chip. In the red circle is depicted the graphene channel that recorded the neuronal action potentials presented in (c). In the live-dead image, it is visible that a bundle of neurites are going through the graphene area of the GFET. In (c) is given the overall timetrace of the recordings. The first three recordings (black, red and blue) are consecutive and around 5 minutes long in total. The pink part of the timetrace is recorded after chip was cleaned (Terg-A-Zyme overnight) in order to eliminate the cells and prove biological origin of the signals. Indeed after the cleaning no APs are visible.

Filters, noise and SNR estimations.

In Supplementary Fig. S11 a timetrace from one HL-1 recording is presented, and the noise and SNR values computed using different methods are presented in Supplementary Table S4. From the table it is visible that the choice of a noise definition influences the level of noise and therefore the final representation of SNR. Choosing RMS instead of 2*MAD one can increase the presented SNR value 3-4 fold.

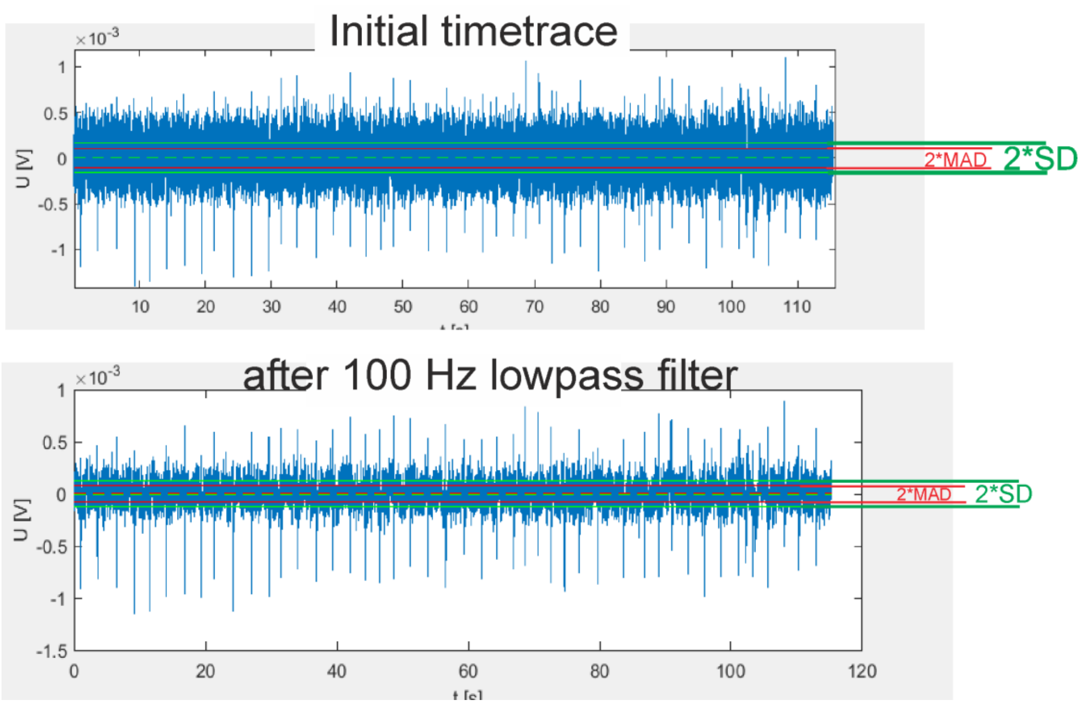

Supplementary Figure S11. The timetrace of HL-1 activity recorded by a GFET. In the top part, the timetrace is shown without an applied filter, while below the 100Hz low pass filter is applied.

Supplementary Table S4. The noise values from the timetraces using different definitions of noise and before and after the 100 Hz low pass filter. The noise and spike amplitude values are given in Volts, spike amplitudes are peak-to-peak values.

			**Noise**						
			MAD	SD	2*MAD	2*SD	RMS		
		initial	1.03E-04	1.61E-04	2.06E-04	3.22E-04			
		100Hz	7.57E-05	1.23E-04	1.51E-04	2.46E-04	9.45E-05		
				**Spikes amplitude**	**Amplitude +-**				
				**1.75E-03**	**1.50E-04**				
				**1.75E-03**	**1.50E-04**				
**SNR**									
MAD		SD		2*MAD		2*SD		RMS	
16.99	1.46	10.87	0.93	8.50	0.73	5.43	0.47		
23.12	1.98	14.23	1.22	11.56	0.99	7.11	0.61	18.52	1.59

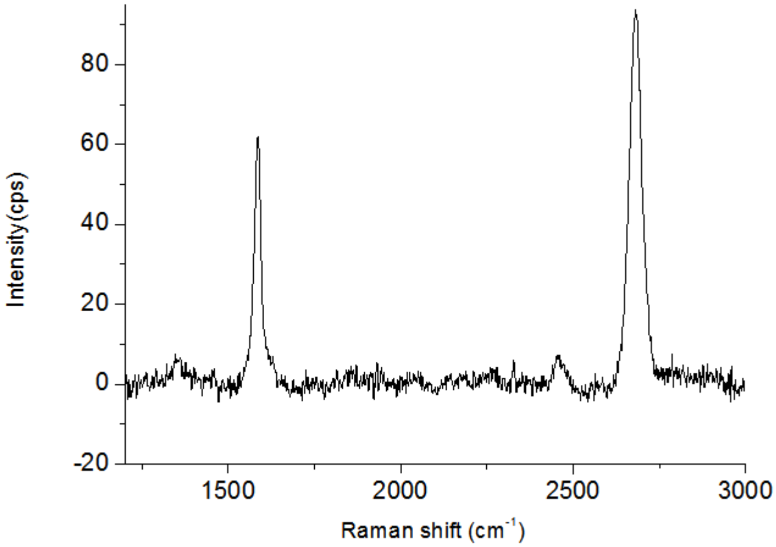

Supplementary Figure S12. Raman spectra of the CVD grown graphene used in this work.

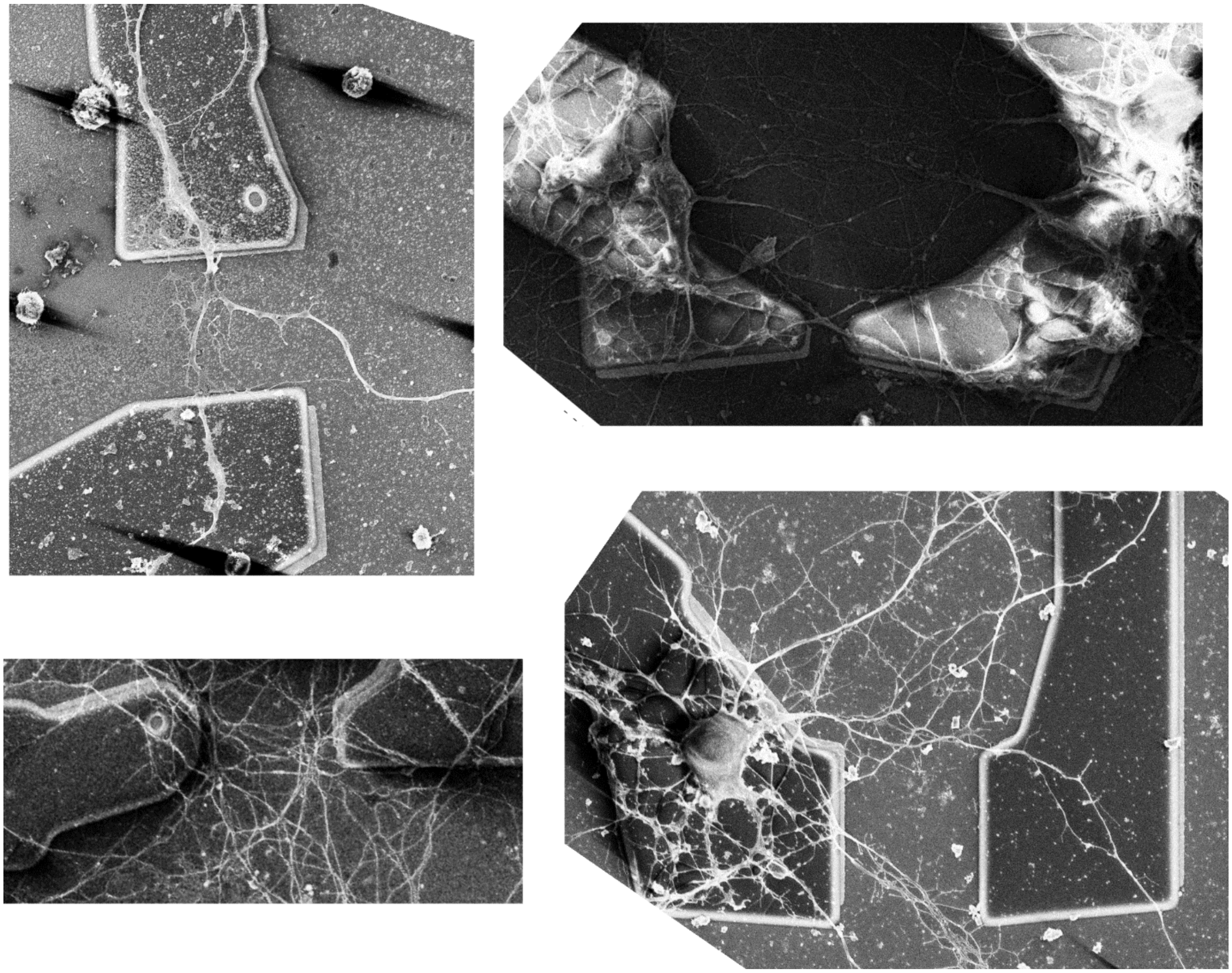

Supplementary Figure S13. More SEM images of the neurons growing on top of our feedline follower passivated GFET chips.

Supplementary References

[S1] Xia, J.; Chen, F.; Li, J.; Tao, N. Measurement of the Quantum Capacitance of Graphene. Nat Nanotechnol 2009, 4, 505–509.

[S2] Hess, L. H.; Hauf, M. V.; Seifert, M.; Speck, F.; Seyller, T.; Stutzmann, M.; Sharp, I. D.; Garrido, J. A. High-Transconductance Graphene Solution-Gated Field Effect Transistors. Appl. Phys. Lett. 2011, 99, 033503.

[S3] Uesugi, E.; Goto, H.; Eguchi, R.; Fujiwara, A.; Kubozono, Y. Electric Double-Layer Capacitance between an Ionic Liquid and Few-Layer Graphene. *Sci. Rep.* **2013**, *3*, 1595.

[S4] Lloret, N.; Frederiksen, R. S.; Møller, T. C.; Rieben, N. I.; Upadhyay, S.; De Vico, L.; Jensen, J. H.; Nygård, J.; Martinez, K. L. Effects of Buffer Composition and Dilution on Nanowire Field-Effect Biosensors. *Nanotechnology* **2013**, *24*, 035501.

[S5] Schwierz, F. Graphene Transistors. Nat. Nanotechnol. 2010, 5, 487–496. [S6] S. Luryi, *Appl. Phys. Lett.* **1988**, 52, 501.

[S7] Hess, L. H.; Seifert, M.; Garrido, J. a. Graphene Transistors for Bioelectronics. *Proc. IEEE* **2013**, *101*, 1780–1792.

[S8] Israelachvili, J. N. Electrostatic Forces between Surfaces in Liquids. In Intermolecular and Surface Forces; Elsevier: Oxford, 2011; pp. 291–340.

[S9] Smith, A. D.; Vaziri, S.; Rodriguez, S.; Östling, M.; Lemme, M. C. Large Scale Integration of Graphene Transistors for Potential Applications in the Back End of the Line. *Solid. State. Electron.* **2015**, *108*, 61–66.
